# Identification of 38 novel loci for systemic lupus erythematosus and genetic heterogeneity between ancestral groups

**DOI:** 10.1038/s41467-021-21049-y

**Published:** 2021-02-03

**Authors:** Yong-Fei Wang, Yan Zhang, Zhiming Lin, Huoru Zhang, Ting-You Wang, Yujie Cao, David L. Morris, Yujun Sheng, Xianyong Yin, Shi-Long Zhong, Xiaoqiong Gu, Yao Lei, Jing He, Qi Wu, Jiangshan Jane Shen, Jing Yang, Tai-Hing Lam, Jia-Huang Lin, Zhi-Ming Mai, Mengbiao Guo, Yuanjia Tang, Yanhui Chen, Qin Song, Bo Ban, Chi Chiu Mok, Yong Cui, Liangjing Lu, Nan Shen, Pak C. Sham, Chak Sing Lau, David K. Smith, Timothy J. Vyse, Xuejun Zhang, Yu Lung Lau, Wanling Yang

**Affiliations:** 1 Department of Paediatrics and Adolescent Medicine, The University of Hong Kong, Hong Kong, China; 2 Department of Pediatric Surgery, Guangzhou Institute of Pediatrics, Guangdong Provincial Key Laboratory of Research in Structural Birth Defect Disease, Guangzhou Women and Children’s Medical Center, Guangzhou Medical University, Guangzhou, China; 3 Department of Rheumatology, The Third Affiliated Hospital of Sun Yat-Sen University, Guangzhou, China; 4 The Hormel Institute, University of Minnesota, Austin, USA; 5 Division of Genetics and Molecular Medicine, King’s College London, London, UK; 6 Department of Dermatology, No.1 Hospital, Anhui Medical University, Hefei, China; 7 Guangdong Provincial Key Laboratory of Coronary Heart Disease Prevention, Guangdong Provincial People’s Hospital, Guangzhou, China; 8 Department of Clinical Biological Resource Bank, Guangzhou Institute of Pediatrics, Guangzhou Women and Children’s Medical Center, Guangzhou, China; 9 School of Public Health, The University of Hong Kong, Hong Kong, China; 10 Radiation Epidemiology Branch, Division of Epidemiology and Genetics, National Cancer Institute, National Institutes of Health, Bethesda, USA; 11 Shanghai Institute of Rheumatology, Renji Hospital, Shanghai Jiao Tong University School of Medicine, Shanghai, China; 12 Department of Pediatrics, Union Hospital Affiliated to Fujian Medical University, Fuzhou, China; 13 Department of Rheumatology, Affiliated Hospital of Jining Medical University, Jining, China; 14 Department of Endocrinology, Affiliated Hospital of Jining Medical University, Jining, China; 15 Department of Medicine, Tuen Mun Hospital, Hong Kong, China; 16 Department of Dermatology, China-Japan Friendship Hospital, Chaoyang, China; 17 Department of Psychiatry, The University of Hong Kong, Hong Kong, China; 18 Department of Medicine, The University of Hong Kong, Hong Kong, China; 19 Shenzhen Institute of Research and Innovation, The University of Hong Kong, Hong Kong, China

**Keywords:** Genome-wide association studies, Autoimmunity, Autoimmune diseases, Systemic lupus erythematosus

## Abstract

Systemic lupus erythematosus (SLE), a worldwide autoimmune disease with high heritability, shows differences in prevalence, severity and age of onset among different ancestral groups. Previous genetic studies have focused more on European populations, which appear to be the least affected. Consequently, the genetic variations that underlie the commonalities, differences and treatment options in SLE among ancestral groups have not been well elucidated. To address this, we undertake a genome-wide association study, increasing the sample size of Chinese populations to the level of existing European studies. Thirty-eight novel SLE-associated loci and incomplete sharing of genetic architecture are identified. In addition to the human leukocyte antigen (HLA) region, nine disease loci show clear ancestral differences and implicate antibody production as a potential mechanism for differences in disease manifestation. Polygenic risk scores perform significantly better when trained on ancestry-matched data sets. These analyses help to reveal the genetic basis for disparities in SLE among ancestral groups.

## Abstract (web summary)

The presentation of systemic lupus erythematosus has been known to differ by ancestry, but the underlying genetic factors remain unclear. Here, the authors report ancestry-specific susceptibility loci and better risk prediction when using data from matched ancestral groups.

## Introduction

Systemic lupus erythematosus (SLE; OMIM 152700) is an autoimmune disease characterized by production of autoantibodies and multiple organ damage. Genetic factors play a key role in the disease, with estimates of its heritability ranging from 43% to 66% across populations^1–3^. Differences in the expression of the disease across ancestral groups have been reported with non-European populations showing an earlier age of onset, 2–4 fold higher prevalence and 2–8 fold higher risk of developing end-stage renal disease than European populations^4–7^. Responses to treatment of SLE with the novel monoclonal antibody against B-cell activating factor (BAFF), Belimumab, also show variation across ancestral groups^8,9^. These findings highlight the heterogeneous nature of the disease, so closer examination of ancestral group differences is likely to improve disease risk prediction and lead to more precise treatment options.

More than 90 loci have been shown to be associated with SLE through genome-wide association studies (GWAS)^10–12^. Trans-ancestral group studies conducted previously were primarily designed to increase power and to identify SLE susceptibility loci shared across ancestries^13,14^. However, due to inadequate power in studies involving non-Europeans, current findings are biased towards loci associated with SLE in European populations. Some risk alleles reported from studies on European populations, such as those in or near *PTPN22, NCF2, SH2B3*, and *TNFSF13B*, are absent in East Asian populations^15^ while a missense variant (rs2304256) in *TYK2* points to a European-specific disease association^16–18^.

The basis for ancestral group differences in the manifestation of SLE at the genome level remains poorly understood. Further studies on non-European populations will help define the genetic architecture underlying SLE and the consequences of patients’ ancestral backgrounds. To this end, we genotyped 8252 participants of Han Chinese descent recruited from Hong Kong (HK), Guangzhou (GZ) and Central China (CC), and combined these data with previous datasets to give a total of ten SLE genetic cohorts consisting of 11,283 cases and 24,086 controls. The increased sample size, particularly for those of Chinese ancestry, allowed identification of novel disease loci and comparative analyses of the genetic architectures of SLE between major ancestral groups. In this work, we identify 38 novel loci associated with SLE and demonstrate both shared and specific genetic components between East Asians and Europeans.

## Results

### Data set preparation

Han Chinese data: After removing individuals with a low genotyping rate or hidden relatedness, the 7596 subjects of Han Chinese descent from HK, GZ, and CC genotyped in this study and the 5057 subjects from the existing Chinese GWAS^13^ gave a Chinese ancestry data set of 4222 SLE cases and 8431 controls (Supplementary Tables 1–2 and Supplementary Fig. 1). Ethnic European Data: Existing GWAS data from European populations^19^ were reanalyzed, based on principal components (PC) matching those for subjects from the 1000 Genomes Project to minimize the potential influence of population substructures^20^ (see “Methods” section) and grouped into three cohorts, EUR GWAS 1–3 (Supplementary Fig. 2). The recent GWAS^21^ data from Spain (SP) was included. After quality control, the European data included 4576 cases and 8039 controls. A further 2485 SLE cases and 7616 controls were included as summary statistics from an Immunochip study of East Asians^22^ (Supplementary Table 2).

### Ancestral correlation of SLE

**Fig. 1:**
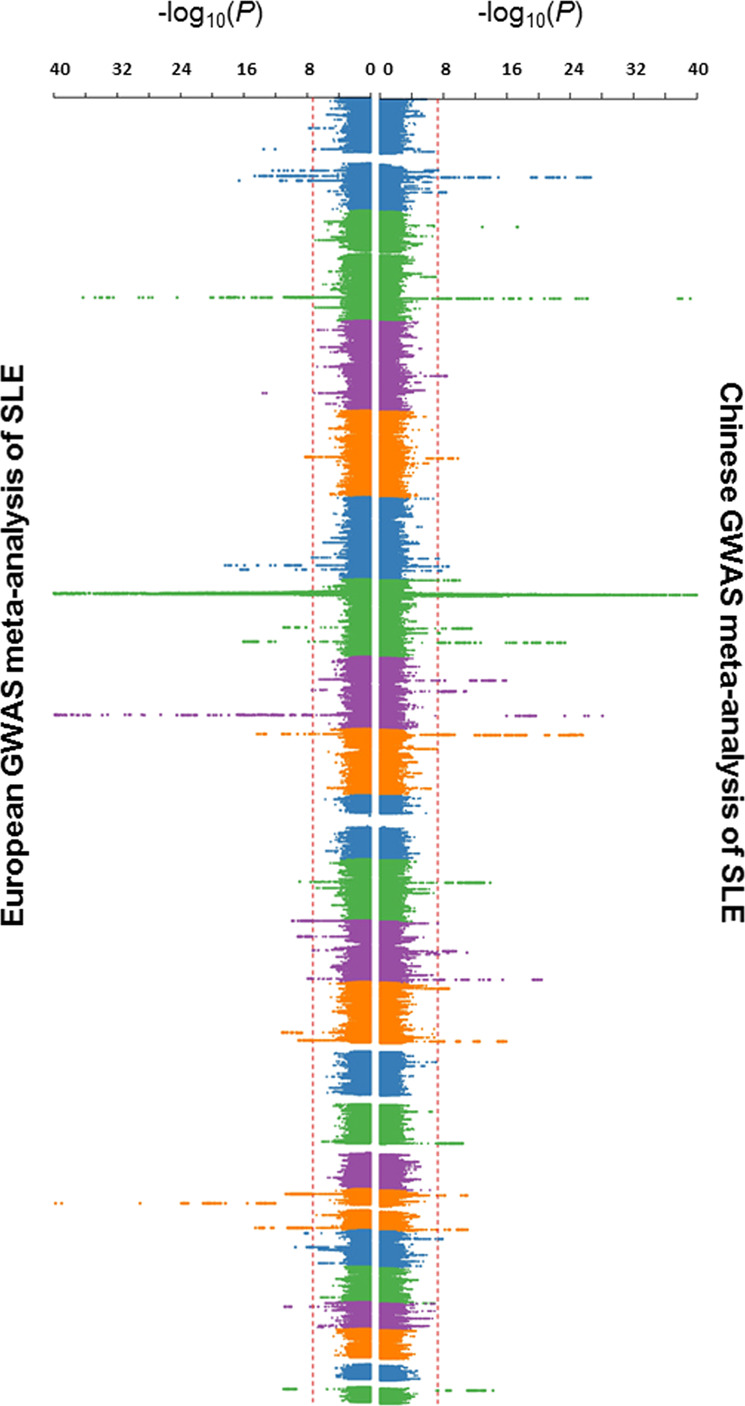
Manhattan plots for association results of systemic lupus erythematosus (SLE) in Chinese and European populations. The Chinese SLE GWAS comprised 4222 cases and 8431 controls and the European GWAS comprised 4576 cases and 8039 controls. The *X*-axis is the *P*-value of association (logistic regression; additive model; two-sided test), as −log_10_ (*P*), for the meta-analyses of the Chinese (right) and European (left) ancestries. Red dashed lines indicate the threshold of genome-wide statistical significance (*P* = 5E − 08). SNPs with *P* < 1E − 40 in an associated locus are not shown from the plot.

Genotype imputation and association analysis were performed independently for each GWAS cohort (Supplementary Figs. 3–4) and as meta-analyses of each ancestral group (Fig. 1; see “Methods” section). The trans-ancestral genetic-effect correlation, *r*_ge_, between the Chinese and European GWAS was estimated to be 0.64 with a 95% confidence interval (CI) of 0.46 to 0.81 by Popcorn^23^ (see “Methods” section), indicating a significant, but incomplete, correlation of the genetic factors for SLE between the two ancestries. This analysis was repeated by removing variants in the human leukocyte antigen (HLA) region (chr6: 25–35 mbp), and the *r*_ge_ increased to 0.78 as a result, suggesting greater ancestral differences for the HLA region.

### Novel SLE susceptibility loci

Meta-analyses, involving a total of 35,369 participants (see “Methods” section; Supplementary Table 2) were conducted. Of the 94 previously reported SLE associated variants (Supplementary Data 1), 59 (62.8%) surpassed a genome-wide significance *P-*value threshold (5.0E−08) and 84 (89.4%) exceeded the threshold of 5E−05 in our study. Thirty-four novel variants reached genome-wide significance and four variants had *P-*values approaching this threshold based on either ancestry-dependent or trans-ancestral meta-analyses. The newly identified loci included the immune checkpoint receptor *CTLA4*, the TNF receptor-associated factor *TRAF3* and the type I interferon gene cluster on 9p21 (Table 1 and Supplementary Data 2). The new loci bring the total of SLE-associated loci to 132 and produce a 23.5% and 16.5% increase in the proportion of heritability explained for East Asians and Europeans, respectively (see “Methods” section).

**Table 1:** Summary association statistics of newly identified SLE-associated variants.

RSid	CHR	Pos (hg19)	AA^a^	AB^a^	Gene context	Annotation	EAS		EUR		Trans		Cochran’s Q-test *P*
							OR^b^	*P*	OR^b^	*P*	OR^b^	*P*	
rs12093154	1	1178925	G	A	*C1QTNF12*	Missense	0.84	4.12E−06	0.82	1.38E−04	0.84	2.51E−09	0.648
rs3795310	1	8431607	C	T	*RERE*	Intronic	0.88	1.40E−03	0.88	8.03E−06	0.88	3.36E−08	0.92
rs28411034	1	38276997	G	A	*MTF1*	UTR3	0.86	5.80E−06	0.87	5.93E−06	0.86	1.50E−10	0.86
rs6702599	1	67825399	A	C	*IL12RB2*	Intronic	0.88	3.56E−02	0.82	1.78E−08	0.84	3.18E−09	0.302
rs1016140	1	117076547	G	T	*CD58*	Intronic	0.87	2.95E−10	1	9.62E−01	0.89	1.26E−08	0.007
rs11264750	1	157497160	A	G	*FCRL5*	Intronic	0.75	1.95E−12	0.81	1.81E−01	0.75	8.70E−13	0.655
rs12132445	1	170811799	G	A	*PRRX1, MROH9*	Intergenic	1.17	6.75E−08	1	9.67E−01	1.08	1.74E−04	1.05E−04
rs549669428	1	174895022	T	G	*RABGAP1L*	Intronic	0.75	1.85E−05	0.87	1.67E−04	0.84	4.53E−08	0.062
rs1547624	1	192543837	A	T	*RGS21, RGS1*	Intergenic	1.17	4.55E−08	1.06	1.38E−01	1.13	1.95E−07	0.025
rs2381401	2	144020974	C	T	*ARHGAP15*	Intronic	1.18	1.11E−07	1.11	1.97E−03	1.15	1.73E−09	0.221
rs9630991	2	191432139	G	A	*NEMP2, NAB1*	Intergenic	0.85	3.34E−06	0.85	6.73E−09	0.85	1.08E−13	0.971
rs11679484	2	198921604	C	A	*PLCL1*	Intronic	1.15	5.59E−05	1.1	1.91E−04	1.12	6.26E−08	0.413
rs3087243	2	204738919	G	A	*CTLA4*	Downstream	0.88	6.44E−06	0.91	6.61E−04	0.89	2.29E−08	0.396
rs438613	3	28072086	T	C	*LINC01980, CMC1*	Intergenic	1.13	6.46E−08	1.07	1.98E−02	1.1	1.32E−08	0.201
rs4690055	4	2748663	G	A	*TNIP2*	Intronic	0.91	4.14E−05	0.91	4.73E−04	0.91	5.28E−08	0.776
rs4697651	4	10721433	C	T	*CLNK, MIR572*	Intergenic	0.85	4.49E−05	0.9	3.26E−04	0.88	6.36E−08	0.256
rs6871748	5	35885982	T	C	*IL7R, CAPSL*	Intergenic	0.88	1.44E−05	0.9	6.40E−04	0.89	3.96E−08	0.579
rs6927090	6	252145	G	T	*DUSP22, IRF4*	Intergenic	1.23	3.48E−10	\	\	\	\	\
rs9387400	6	116694120	C	A	*DSE*	Intronic	0.74	3.14E−08	0.94	2.96E−02	0.89	5.89E−06	1.17E−04
rs13260060	8	71218360	G	A	*NCOA2*	Intronic	0.89	1.92E−08	0.99	7.55E−01	0.9	7.94E−08	0.044
rs2445610	8	128197088	A	G	*PRNCR1, CASC19*	Intergenic	0.89	5.26E−08	0.96	1.82E−01	0.91	2.60E−07	0.027
rs7815944	8	129427518	A	G	*LINC00824*	Intronic	0.86	1.78E−09	0.94	4.27E−01	0.87	2.21E−09	0.303
rs4978037	9	21228423	T	C	*IFNA17*	Upstream	1.12	7.36E−05	1.12	3.92E−04	1.12	5.68E−08	0.902
rs1405209	9	102585545	T	C	*NR4A3*	Intronic	1.11	3.10E−04	1.12	8.55E−05	1.11	2.42E−08	0.816
rs4745876	10	64425126	G	A	*ZNF365*	Intronic	0.88	1.36E−06	0.9	1.40E−02	0.88	3.66E−08	0.569
rs10999979	10	73501066	C	A	*CDH23*	Intronic	1.16	1.01E−07	1.28	3.61E−01	1.17	4.65E−08	0.726
rs7975703	12	121099302	C	T	*CABP1*	Intronic	0.87	1.47E−06	0.91	3.89E−03	0.88	2.99E−08	0.367
rs76725306	13	50177453	G	A	*RCBTB1, ARL11*	Intergenic	1.15	1.38E−06	1.19	8.22E−03	1.16	3.60E−08	0.617
rs1885889	13	100091300	A	G	*UBAC2*,	Intergenic	0.87	7.93E−10	0.86	5.69E−05	0.87	2.05E−13	0.87
rs12148050	14	103263788	A	G	*TRAF3*	Intronic	0.93	1.04E−03	0.87	1.11E−06	0.91	2.57E−08	0.062
rs869310	15	77830306	T	G	*LINGO1*	Intergenic	0.86	1.11E−05	0.9	2.54E−04	0.88	1.90E−08	0.294
rs9899849	17	7234983	G	A	*NEURL4, ACAP1*	Intergenic	0.97	3.32E−01	1.21	1.10E−08	1.08	1.08E−03	1.72E−06
rs2384991	19	18386634	A	C	*JUND*	Intergenic	1.1	2.88E−05	1.12	4.22E−04	1.11	4.95E−08	0.657
rs13344313	19	18517767	G	A	*LRRC25, SSBP4*	Intergenic	0.86	1.35E−05	0.86	2.33E−06	0.86	1.34E−10	0.959
rs405858	19	33106621	C	T	*ANKRD27*	Synonymous	0.87	1.70E−08	0.93	1.14E−02	0.89	2.38E−09	0.108
rs3760667	19	49814832	C	T	*SLC6A16*	Intronic	0.86	1.38E−06	0.88	3.73E−04	0.87	2.31E−09	0.595
rs10419308	19	55739813	G	A	*TMEM86B*	Intronic	0.89	3.60E−02	0.83	2.12E−07	0.84	3.60E−08	0.326
rs6074813	20	1541752	G	T	*SIRPD, SIRPB1*	Intergenic	1.12	8.49E−05	1.12	1.01E−04	1.12	3.23E−08	0.93

^a^AA: allele A, AB: allele B.

^b^Odds ratio (OR) is with respect to allele B (allele B would be the risk allele when OR >1). Association results for each dataset are available in Supplementary Data Table 4. Cochran’s Q test was used to assess differences in effect size estimates between EAS and EUR groups. SNP rs6927090 failed quality control in the European GWAS due to very low minor allele frequency and low quality on imputation.

### Annotation of SLE susceptibility loci

Functional annotations that might be enriched with SLE susceptibility loci were evaluated by the stratified LD score regression method^24^ (see “Methods” section). For non-cell type-specific annotations, heritability was significantly enriched in transcription start sites (*P* = 2.47E−05), regions that are conserved in mammals (*P* = 8.22E−03) and ubiquitous enhancers that are marked with H3K27ac or H3K4me1 modifications (*P* = 4.43E−02, *P* = 5.03E−02, respectively; Supplementary Fig. 5). Based on H3K4me1 modifications (associated with active enhancers) across 127 cell types, enrichment of specific cell types was investigated (see “Methods” section). Cells that surpassed the false discovery threshold rate (FDR < 0.05) were mostly hematological cells, with B and T lymphocytes the most prominent cell types associated with SLE (Supplementary Fig. 6a). Similar results were observed based on H3K4me3 modifications (associated with promoters of active genes) (Supplementary Fig. 6b).

We used the Regulatory Element Locus Intersection (RELI) method^25^ to identify transcription factors (TFs) whose binding sites are enriched in SLE-associated loci. Out of 1544 ChIP-seq datasets with a total of 344 TFs in 221 cell types, 249 datasets showed significant enrichment with the associated loci (corrected *P* < 1.00E−05; see “Methods” section; Supplementary Data 3). Consistent with results from previous studies^25,26^, the associated SNPs were strongly intersected with binding sites of immune-related TFs, including NFATC1, NF-κB, STAT5A, IRF4, and viral protein EBNA2.

### Identification of putative disease genes and pathways

Excluding the HLA region, 179 putative disease genes were identified across the disease-associated loci reported before and those newly identified in this study (Supplementary Data 4; see “Methods” section). A significant level of protein-protein connectivity corresponding to genes found at the novel loci and known SLE-associated loci was observed (*P* < 1E−16; Supplementary Fig. 7; see “Methods” section). Forty-five pathways were significantly enriched with these putative SLE susceptibility genes (ToppGene^27^, FDR < 0.05; Supplementary Table 3). The pathways of cytokine signaling, IFN-α/β signaling, Toll-like receptor (TLR) signaling, and B and T cell receptor signaling showed greatest enrichment. The RIG-I-like receptor signaling (*P* = 5.83E−10) and TRAF6-mediated IRF7 activation (*P* = 6.41E−10) pathways were designated as SLE associated pathways primarily based on genes newly identified in this study.

### Trans-ancestral fine-mapping of disease-associated loci

**Fig. 2:**
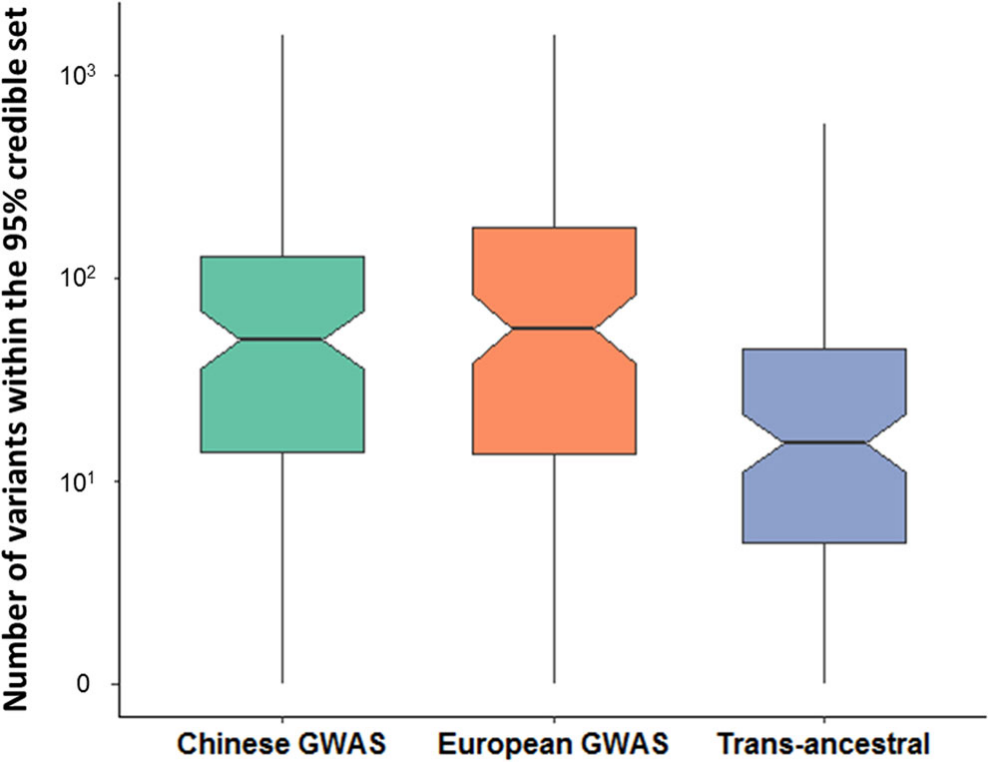
Fine-mapping across 108 SLE-associated loci based on the association results from the Chinese SLE GWAS, the European GWAS, and the trans-ancestral meta-analyses. The *Y*-axis indicates the number of potential causal variants at each locus based on the 95% credible sets of the association results from the Chinese SLE GWAS (green), the European GWAS (orange) and trans-ancestral meta-analyses (purple). The upper and lower bounds of the boxes represent the first and the third quartiles, respectively, and the central lines indicate the median. The two lines outside the box extend to the highest and lowest observations. *N* = 108 independent loci associated with SLE for each category.

One hundred and eight SLE-associated loci tagged by SNPs having a minor allele frequency (MAF) greater than 1% in both Chinese and European populations were examined by PAINTOR^28^, making use of the differences in LD between ancestries. The median number of putative causal variants in the 95% credible sets reduced from 57 per locus when using only the European GWAS to 16 per locus when using data from both ancestries (one-sided paired *t*-test *P* = 9.79E−07, Fig. 2). The number of disease-associated loci with five or fewer putative causal variants increased from four when using the European GWAS alone to 15 (Supplementary Data 5). A single putative causal variant was identified for the *WDFY4* and *TNFSF4* loci, the latter of which was functionally validated in a previous study^29^ (Supplementary Fig. 8).

### Ancestral group differences

**Fig. 3:**
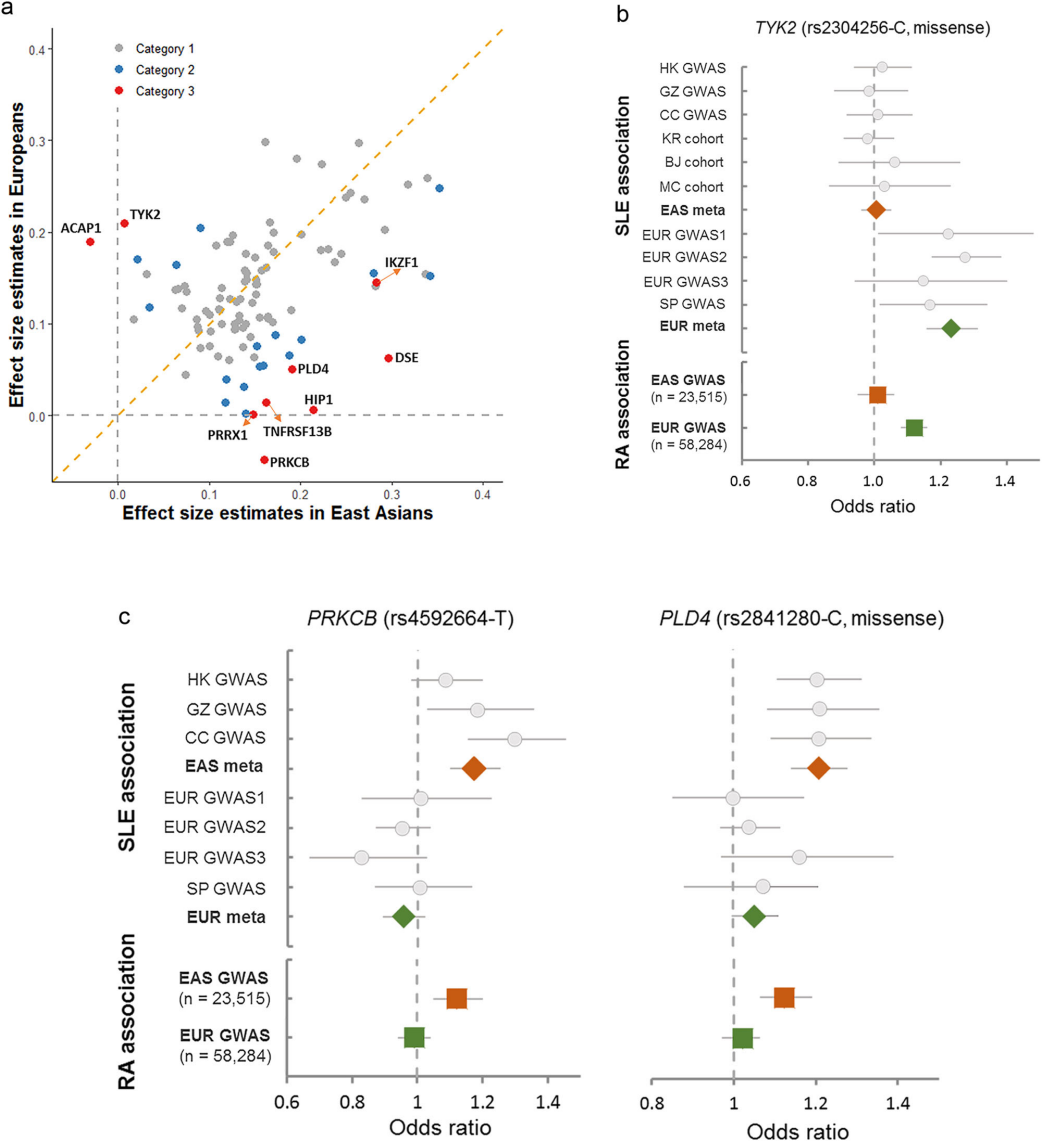
Genetic loci showing significant ancestral differences in effect-size estimates for systemic lupus erythematosus (SLE) and rheumatoid arthritis (RA). **a** Correlation of effect-size estimates for SLE between East Asian (*X*-axis) and European (*Y*-axis) populations (*r* = 0.58, two-sided *P*-value = 6.52E−11). Disease-associated variants with FDR adjusted CQ-test *P*-value <0.05 (category 3) are labeled in red, and the variants with CQ-test *P*-value < 0.05 but FDR adjusted CQ-test *P*-value ≥0.05 (category 2) are labeled in blue. **b**, **c** Forest plots of association from each cohort at the *TYK2*, *PRKCB* and *PLD4* loci. Diamonds represent the combined estimates of odds ratio (OR) in East Asians (EAS, red) and Europeans (EUR, green) for association with SLE. Standard error bars of OR represent 95% confidence intervals of the estimates. Squares represent OR estimates for rheumatoid arthritis (RA) in EAS (red) and EUR (green) populations. Regional plots for each locus are available in Supplementary Fig. 10. HK, Hong Kong; GZ, Guangzhou; CC, Central China; KR, Korean; BJ, Beijing; MC, Malaysian Chinese; SP, Spanish.

Based on the analysis of Cochran’s Q (CQ)-test that assesses heterogeneity of effect-size estimates from different ancestral groups, SLE-associated variants or loci outside of the HLA region were divided into four categories: (1) ancestry-shared disease loci tagged by variants with CQ-test *P* ≥ 0.05; (2) putative ancestry-heterogeneous disease loci with CQ-tests of *P* *<* 0.05 but FDR adjusted CQ-test *P* ≥ 0.05; (3) ancestry-heterogeneous disease loci with FDR adjusted CQ-test *P* *<* 0.05; and (4) disease loci tagged by associated variants with the risk allele absent in one of the two ancestries^15^ (Supplementary Table 4). Nine disease variants, other than those absent or rare (MAF < 0.01) in one of the two ancestries^15^, showed significant differences in effect-size estimates between the two ancestral groups and were considered ancestry-heterogeneous (FDR adjusted CQ-test *P* < 0.05, category 3; Fig. 3a and Supplementary Table 5). Within this category, variants in the *HIP1*, *TNFRSF13B*, *PRKCB*, *PRRX1*, *DSE,* and *PLD4* loci were associated with SLE only in East Asians and variants in *TYK2* and *NEURL4-ACAP1* only in Europeans (*P* < 5.0E−08 in one ancestry but *P* > 0.01 in the other, with non-overlapping of the 95% CIs of the ORs). These eight loci were thus considered ancestry-specific. SNP rs4917014, a variant near *IKZF1*, showed a significantly stronger effect in East Asians (OR = 1.33, *P* = 5.18E−29) than in Europeans (OR = 1.16, *P* = 1.34E−06; CQ-test *P* = 4.02E−04). These findings were supported by analyses in each cohort (Supplementary Figs. 9–10).

On reanalyzing data from association studies on rheumatoid arthritis (RA)^30^, the variants in the *PRKCB* and *PLD4* loci were found to be associated with RA in East Asians but not in Europeans (CQ-test *P* = 0.004 and 0.010 for *PRKCB* and *PLD4*, respectively), while the variant in *TYK2* was found associated in Europeans only (CQ-test *P* = 0.002; Fig. 3b and c). This consistency with the differences found in the SLE study suggests that shared mechanisms could be responsible for ancestral group differences among autoimmune diseases.

### Colocalization of loci across ancestral groups

Colocalization methods consider many SNPs, rather than only the leading variant in a locus, to compare association signals between ancestral groups. The ancestry-shared disease loci showed much higher posterior probabilities (PP) of colocalization (mean of PP = 0.42) than the ancestry-specific loci (mean of PP = 0.03; Supplementary Fig 11a). For example, the *MTF1*, *IKBKE* and *TNIP1* loci in category 1 showed strong posterior probabilities (≥90%) of colocalization under a Bayesian test^31^ (see “Methods” section), suggesting shared causal effects between the two ancestries. The eight ancestry-specific loci in category 3 showed low posterior probabilities of colocalization (1–10%), consistent with the CQ-test of the top variant of each locus (above and Supplementary Fig. 10).

Since LD differences between ancestries may affect the colocalization results, we compared the SLE association signals from the Chinese populations with those on 27 non-immune-related phenotypes studied in Europeans (Supplementary Table 6) to serve as colocalization baseline values. While posterior probabilities for colocalization at the ancestry-shared *MTF1*, *IKBKE* and *TNIP1* loci were much greater than the baseline values, there were no differences for the six Asian-specific disease loci (Supplementary Fig. 11b), thereby excluding the potential influence of LD. The European-specific loci (*TYK2* and *NEURL4-ACAP1*) were not evaluated this way due to lack of public data.

### Functional annotation of the ancestry-heterogeneous loci

The ancestry-heterogeneous loci appear to be enriched for functions related to antibody production. Two of the nine putative disease genes at ancestry-heterogeneous loci (category 3), *TNFRSF13B* and *IKZF1*, are causal genes for human primary immunodeficiency disorders (PID)^32^ presenting with primary antibody deficiencies (PADs), whereas none of the disease genes at the putative ancestry-heterogeneous loci (category 2, 0/22; Fisher exact test *P* = 0.07) or the ancestry-shared loci (category 1, 0/120; Fisher exact test *P* = 0.004) are known to cause PADs in humans. Two of the East Asian-specific disease variants, those in the *TNFRSF13B* and *PRKCB* loci, were associated with serum immunoglobulin levels in East Asian populations^33–35^, whereas none of the variants in loci belonging to category 1 and 2 were found to be associated.

*TNFRSF13B*, which encodes a BAFF receptor, TACI, plays a major role for immunoglobulin production^36–38^. In this study, a missense variant in *TNFRSF13B*, rs34562254, was specifically associated with SLE in Chinese populations (OR = 1.18, *P* = 2.88E−08 in Chinese; OR = 1.01, *P* = 0.75 in Europeans). In European populations, an SLE-associated variant in the 3’-UTR of *TNFSF13B* (encoding BAFF), which is absent in Asian populations (category 4), was associated with serum levels of total IgG, IgG1, IgA, and IgM^39^.

Mice deficient in the orthologs of four of the nine putative disease genes (44.4%) at the ancestry-heterogeneous loci for SLE, *Tnfrsf13b-*, *Ikzf1-*, *Prkcb-,* and *Tyk2*-demonstrated abnormal IgG levels^40^ (MP:0020174), while at putative ancestry-heterogeneous loci (4/22 or 18.2%; OR = 3.43, Fisher exact test *P* = 0.185) or ancestry-shared loci (14/120 or 11.6%; OR = 5.92, Fisher exact test *P* = 0.027), proportionately fewer genes caused aberrant IgG levels in mice. Orthologs of four of the twelve (33.3%) putative disease genes from disease loci where the risk allele is monomorphic in one of the ancestries (*PTPN22, TNFSF13B, IKZF3*, and *IGHG1*; category 4) also demonstrated abnormal immunoglobulin production in gene knockout mouse models^36,39,41–43^.

### Evolutionary signatures for the disease loci

Disease loci with heterogeneity between East Asians and Europeans might have undergone differential selection pressures in recent human history, as has been shown for the SLE risk variant in *TNFSF13B*^39^. Frequency variances, as fixation indexes (*F*_st_), for the variants of the first three categories were calculated using 3324 controls from the HK cohort and 5379 controls from EUR GWAS 2 cohort (see “Methods” section). Higher *F*_st_ would indicate a larger frequency difference between the two ancestries. Mean *F*_st_ values for the ancestry-shared, putative ancestry-heterogeneous and ancestry-heterogeneous variants were 0.054, 0.061, and 0.084, respectively. Although a small sample, three (*DSE*, *HIP1*, *TNFRSF13B*) of the nine ancestry-heterogeneous variants (33.3%) showed *F*_st_ ≥ 0.15 (empirical *P* < 0.03), while only 10% of the putative ancestry-heterogeneous variants and 8.8% for the ancestry-shared disease variants had *F*_st_ ≥ 0.15 (Supplementary Fig. 12a).

**Fig. 4:**
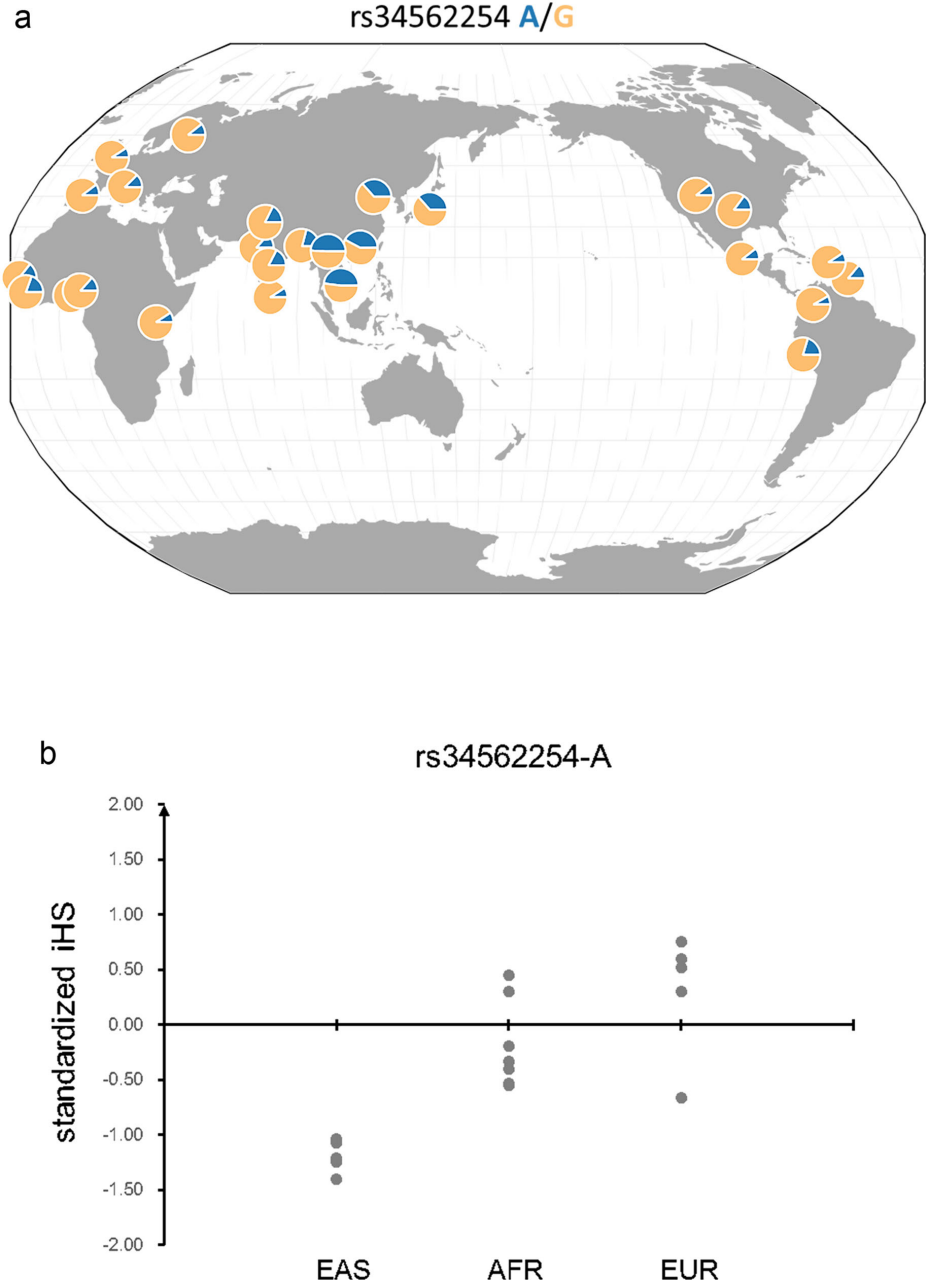
Risk allele frequency and standardized integrated haplotype scores (iHS) across different populations for the Asian-specific variant at *TNFRSF13B* locus. **a** Frequency for the risk allele rs34562254-A across populations. **b** Standardized iHS for the variants across different continental populations (EAS: East Asians; AFR: Africans; EUR: Europeans). The risk allele rs34562254-A is a derived allele, and negative iHS value indicates that the haplotypes carrying the derived allele are longer than the haplotypes carrying the ancestral allele. The frequency and iHS were calculated using data from the 1000 Genomes Project.

In addition, recent positive selection, measured by standardized integrated Haplotype Scores (iHS)^44^, was investigated at the associated loci. A significant correlation of the iHS scores, estimated using control subjects from HK and EUR GWAS 2 cohorts (see “Methods” section), supports recent positive selection for the shared associated variants (categories 1; *r* = 0.28, *P* = 0.03; Supplementary Fig. 12b). This is consistent with results using data from Southern Han Chinese (CHS) and Utah residents of European ancestry^45^ (CEU; *r* = 0.32, *P* = 0.008). However, there was no evidence of such a correlation for disease variants that showed ancestry heterogeneity (category 2 and 3; Supplementary Fig. 12b). For example, in the BAFF system, the derived risk allele rs34562254-A in *TNFRSF13B* is much more prevalent in East Asians than in other populations (Fig. 4a) and has a significantly longer haplotype for the derived risk allele than the ancestral allele (more negative standardized iHS score) in East Asian populations than in Africans (*P* = 3.2E−04) or Europeans (*P* = 4.4E−04) (Fig. 4b), suggesting recent positive selection for the risk allele in East Asians.

### Polygenetic risk scores for SLE and their accuracies across ancestries

Polygenic risk scores (PRS) have been used to estimate individual risk to complex diseases, such as coronary artery disease^46^ and schizophrenia^47^. However, as the majority of GWAS findings used to calculate these scores are based on European populations, their accuracy in other populations may be limited. PRS for SLE, trained by data on European populations, were tested on individuals of three Chinese cohorts using the lassosum algorithm^48^ (see “Methods” section). The area under the receiver-operator curve (AUC) ranged from 0.62 to 0.64 for the three Chinese cohorts. Similar results were observed in the reverse case (Supplementary Fig. 13a). The LDpred^49^ algorithm produced similar results (Supplementary Fig. 13b). These analyses suggest a partial transferability of PRS between the two ancestries.

**Fig. 5:**
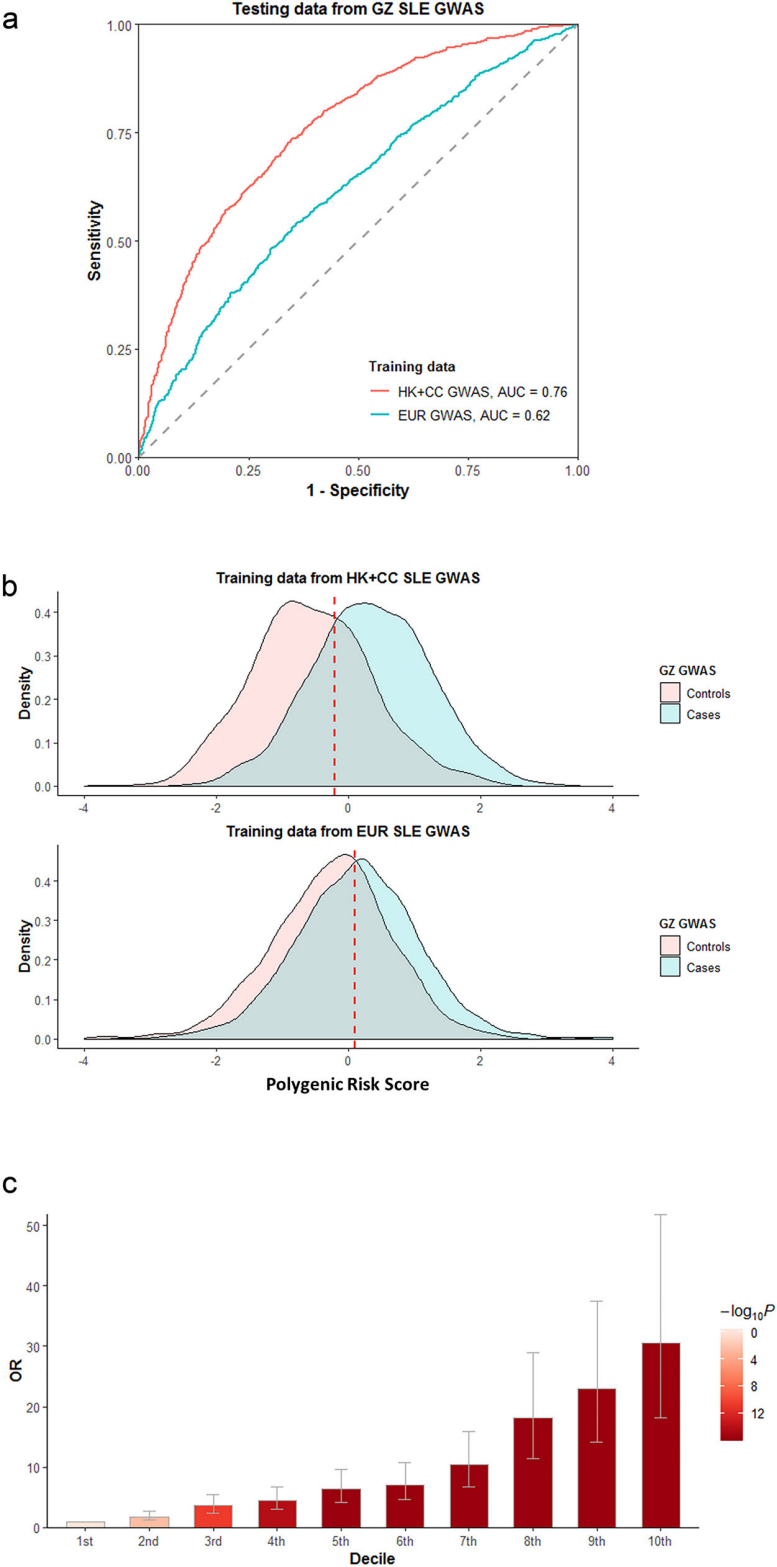
Performance of polygenic risk scores (PRS) calculated by summary statistics from different ancestral groups. **a** Performances of PRS are indicated by area under receiver operating characteristic curve (AUC). PRS for individuals from the Guangzhou (GZ) cohort were calculated using summary statistics from ancestry-matched Chinese populations (2618 cases and 7446 controls; red) and European populations (4576 cases and 8039 controls; blue). **b** Distribution of PRS for SLE cases (blue) and controls (pink) from the GZ cohort. The PRS distribution was estimated using summary statistics from the ancestry-matched Chinese cohorts (upper panel) or from the mismatched European GWAS (lower panel). The optimal threshold for disease risk prediction is indicated by the red vertical line. **c** Odds ratios (ORs) of disease risk across different PRS groups in the GZ GWAS. The samples from GZ are equally divided into ten groups (*n* = 259 independent samples in each group) based on PRS estimated from ancestry-matched data. The 1st decile represents the lowest PRS group while 10th decile refers to the group of samples with the highest PRS. ORs and 95% confidence intervals (bars) for each group were calculated by reference to the 1st decile group.

Using samples from the GZ cohort as the validation dataset, performance of predictors trained using GWAS summary statistics from the HK and CC cohorts (2618 cases and 7446 controls) or from the European cohorts (4,576 cases and 8039 controls) were evaluated. Ancestry-matched predictors significantly outperformed (AUC = 0.76, 95% CI: 0.74–0.78) ancestry mismatched predictors (AUC = 0.62, 95%CI: 0.60–0.64) (Fig. 5a). When the analysis was repeated by randomly choosing the same number of samples (1500 cases and 1500 controls) from each of the Chinese and European GWAS as training data, a similar difference was observed (Supplementary Fig. 14). Ancestry-matched PRS for samples in the GZ cohort had a mean difference of 0.89 (standard deviation) between the SLE case and control groups (*t*-test *P* = 9.01E−116) and disease classification using the optimal threshold achieved 73.4% sensitivity and 65.4% specificity (Fig. 5b; see “Methods” section). Disease risk increased with higher PRS, with individuals in the highest PRS decile having a much higher disease risk than those in the lowest decile (OR = 30.3, Chi-square test *P* = 6.23E−54; Fig. 5c).

## Discussion

As non-European groups appear to be more severely affected by SLE, greater genetic data on these groups are likely to be highly informative. By increasing the number of subjects of East-Asian ancestry to levels roughly equivalent to those of European subjects, and including previously published data, we have made cross ancestral group studies possible.

Through ancestry-dependent and trans-ancestral meta-analyses, we identified 38 novel loci associated with SLE, bringing the total number of SLE-associated loci to 132. High level functional annotation of these SLE associated loci implicated hematological cells, particularly B and T lymphocytes, cytokine signaling and other immune system pathways. Consistent with previous findings^50^, we demonstrated the value of trans-ancestral data in significantly reducing the number of putative causal variants at each disease-associated locus, which may facilitate future functional and mechanistic studies.

There was strong evidence of heterogeneity on SLE associations between the two ancestries, which were not likely to be artefacts of study power. Eight variants (that are common in both ancestral groups) were associated with disease in only one of the ancestral groups. For three of these, a similar ancestral difference was found by re-analyzing association data on RA^30^. This might suggest that common mechanisms account for ancestral differences in autoimmune diseases.

Genes at the ancestry-heterogeneous disease loci seemed more likely to be involved in regulation of immunoglobulins than genes at the ancestry-shared disease loci. Immunoglobulin levels are highly heritable^51^ and have been found to differ between ancestries, with African Americans and Asians having higher serum immunoglobulin levels than people of European ancestry^52–55^. Higher antibody levels in non-European populations might have contributed to their higher prevalence of SLE and further study of intrinsic differences in immune function among ancestries may be informative.

That differential mechanisms may exist for antibody regulation between East Asian and European populations is supported by the association of SLE with the BAFF signaling system. This system, which is part of the initial reaction to host-pathogen interactions^37^, might be under positive selection due to different environmental exposures^37,39^. BAFF (*TNFSF13B*) and its receptors, one of which is TACI (*TNFRSF13B*), play essential roles in B cell survival and differentiation^36–38^. The SLE risk allele in the gene encoding BAFF is completely absent in Chinese populations^39^ and a missense variant in the gene encoding TACI (*TNFRSF13B*) was found to be specifically associated with SLE in East Asians in this study. Both these genes, *TNFSF13B*^39^ in Europeans and *TNFRSF13B* in East Asians, were found to have undergone positive selection in recent human history. Adaptation of the host to resist pathogens may underlie some of the ancestral group-heterogeneity.

TACI is expressed at very low levels in human newborns and mice before exposure to pathogens^56,57^ and previous studies have shown that certain pathogens can ablate B cell responses by modulating the expression of TACI^58–60^. The BAFF risk allele was shown to significantly upregulate humoral immunity^39^, and whether this is the case for the risk allele in TACI should be investigated. TACI blockers, such as Atacicept^61^ and Telitacicept^62^, might give better responses to SLE in patients of Asian ancestry and recent results of a Phase 2b study showed that the Telitacicept was efficacious for SLE patients in China^62^. In addition, the variant found in *TNFRSF13B* may be a useful genetic marker for the prescription of Belimumab and TACI blockers, a hypothesis that may warrant further study.

In addition to gene-environment interactions, gene-gene interactions could be another reason for the population difference. However, such effect requires much bigger power to be detected. Recent study showed that the penetrance of monogenic risk mutations could be dependent on polygenic background^63^, indicating a complex format of gene-gene interactions. Besides, ancestry-dependent tagging of untyped causal variants due to different LD structure may result in artefacts of population-different associations. This is an area that warrants further investigation, which demands both increase of study power and innovative methodologies.

Our analyses have identified a substantial number of novel SLE-associated genetic loci and deepened our understanding of the genetic factors that may underly the differences in the manifestation of SLE between peoples of European and non-European ancestry. Like a recent PRS study in SLE^64^, but to a greater extent, we have shown that PRS achieved a far better performance when based on ancestry-matched populations. Our findings contribute new insights into precise treatments, and to risk prediction and prevention of SLE.

## Methods

### Overview of samples

8252 subjects of Han Chinese descent from Hong Kong (HK), Guangzhou (GZ) and Central China (CC) were genotyped in this study. The institutional review boards of the institutes collecting the samples (The University of Hong Kong, Hospital Authority Hong Kong West Cluster and Guangzhou Women and Children’s Medical Center) approved the study and all subjects gave informed consent. These subjects were genotyped by the Infinium OmniZhongHua-8, the Infinium Global Screening Array-24 v2.0 (GSA) and the Infinium Asian Screening Array-24 v1.0 (ASA) platforms. Illumina GenomeStudio 2.0 was used to perform genotyping for individuals and transformed the results into PLINK format. Fourteen samples were randomly selected and genotyped by different platforms. High concordance rates (>99.9%) were observed for genotypes derived from the different platforms (Supplementary Table 1). Principal component (PC) analysis was performed to examine potential batch effects and no significant differences were observed from the PCs for data genotyped by different platforms and from different batches (Supplementary Fig. 1). Compared to our previous SLE GWAS^13^, 2042 more samples (992 cases and 1050 controls) were added to the HK cohort, and 2917 additional controls were combined with the CC cohort (named AH in the previous study^13^) after quality control procedure. The samples in the GZ cohort were newly recruited and genotyped in this study.

For the European data^13^, we split the samples into three cohorts to better control for population substructures (see the section below). Summary statistics for the GWAS from Spain^21^ (SP) and three ImmunoChip data sets from Korea (KR), Han Chinese in Beijing (BJ) and Malaysian Chinese (MC)^22^ were included in our analyses. In total, 11,283 SLE cases and 24,086 controls were involved in this study, and the sample size for each cohort was summarized in Supplementary Table 2.

### Quality control and association study

Genotype Harmonizer^65^ was used to align the strands of variants of the Chinese GWAS to the reference of the 1000 Genomes Project Phase 3 panel. Variants with a low call rate (<90%), low minor allele frequency (<0.5%) and violation of Hardy–Weinberg equilibrium (*P*-value < 1E−04) were removed. Quality control required the following criteria: (i) missing genotypes were below 5%, (ii) hidden relatedness (identity-by-descent) with other samples was ≤12.5% (iii) inbreeding coefficients with other samples ranged from −0.05 to 0.05, and (iv) not having extreme PC values as computed for individuals using EIGENSTRAT embedded in PLINK^66,67^. After quality control, pre-phasing used SHAPEIT^68^ and individual-level genotype data were imputed to the density of the 1000 Genomes Project Phase 3 reference using IMPUTE2^69^. We compared allele frequencies of all the variants after imputation for the same control groups genotyped by different platforms at different time points, and 190,618 variants were removed from further analyses due to significant differences (*P-*value < 5E−05). For association analysis SNPTEST^70^ was used to fit an additive model. Top PCs and the BeadChip types were included as covariates. The number of PCs to be adjusted for in each analysis was determined using a scree plot with a cutoff when the plot levels off. Variants with imputed INFO scores <0.7 were excluded. The genomic inflation factors (λ_GC_) for the HK, GZ, and CC GWAS were 1.04, 1.03, and 1.04, respectively, and the LD score regression (LDSC)^71^ intercepts were 1.03, 1.02, and 1.03, respectively. Manhattan plots for each cohort are shown in Supplementary Fig. 3.

For the European SLE GWAS data, the λ_GC_ and LDSC intercept listed in LD hub seemed inflated (λ_GC_ = 1.17 and LDSC intercept = 1.10)^20^. Thus, the data were reanalyzed to minimize the potential influence of sub-population stratification (and see below). PCA analysis showed that subjects from the existing European data were more diverse than the Chinese subjects used in this study (Supplementary Fig. 2a). The European individuals were grouped into three cohorts by their PCs relative to the subjects of the 1000 Genomes Project. Subjects in the EUR GWAS 1, EUR GWAS 2, EUR GWAS 3 cohorts shared similar PCs with individuals of Spanish (IBS), northern and western European (CEU and GBR) and Italian (ITS) origins, respectively (Supplementary Fig. 2b and Supplementary Table 2). Quality control, imputation and association analyses were conducted, as for the Chinese datasets, in each cohort. λ_GC_ for the three European GWAS datasets were 1.05, 1.08, and 1.03, respectively, and the LDSC intercepts were 1.03, 1.04, and 1.00, respectively. Manhattan plots for each cohort are shown in Supplementary Fig. 4.

### Meta-analyses of SLE association studies

Meta-analyses for the Chinese and European SLE GWAS were conducted independently. The summary association statistics from HK, GZ and CC GWAS (4222 SLE cases and 8431 controls) were combined in a meta-analysis using a fixed-effect model, weighted by the inverse-variance^72^. The λ_GC_ for the Chinese meta-analysis was 1.09 and the LDSC intercept was 1.04. For the European data, the EUR GWAS 1–3 datasets were combined with the SP GWAS^21^ in the meta-analysis (4576 cases and 8039 controls). λ_GC_ and the LDSC intercept for the European SLE meta-analysis reduced to 1.11 and 1.03, respectively.

Trans-ancestral meta-analysis across the Chinese and European GWAS cohorts used the fixed-effect model. The summary association statistics for the Immunochip data from KR, BJ, and MC^22^ were included as an in silico replication. The λ_GC_ for the trans-ancestry meta-analysis was 1.15, and the LDSC intercepts computed by using the LD score from either East Asian or European panels were 1.06 and 1.08, respectively.

### Genetic correlation between the two ancestries

Trans-ancestral genetic correlation from the meta-analysis results for Chinese (HK, CC, and GZ GWAS) and Europeans (EUR GWAS 1–3 and SP GWAS) were estimated using the Popcorn algorithm^23^ based on common SNPs in the autosomes. The disease prevalence in Chinese and European populations were set to be 1‰ and 0.3‰, respectively^4^. SNPs were removed from this analysis according to the following criteria: (1) SNPs with strand-ambiguities (A/T or C/G alleles); (2) having MAF <5%; and (3) having imputed INFO score <0.9. The cross-ancestry LD scores were estimated using control subjects from the HK cohort (*n* = 3324) and EUR GWAS 2 cohort (*n* = 5379).

### Heritability explained by the SLE-associated variants

The variance in liability explained by the SLE-associated variants was measured using VarExplained program^73^. Variants in the HLA region were excluded in the analysis. The disease prevalence was set to be 1‰ for East Asians and 0.3‰ for Europeans^4^. The novel loci increased the heritability explained from 0.10 to 0.13 for East Asians, and from 0.08 to 0.09 for Europeans.

### Functional annotations of SLE associated SNPs

The stratified LD score regression method^24^ was applied on the trans-ancestral meta-analysis result to partition SNP-heritability across functional annotations. Twenty-eight categories of annotations that are not cell type specific (Supplementary Fig. 5) provided by this source were studied. For cell type-specific analyses, H3K4me1 and H3K4me3 modifications across 127 cell types (Supplementary Fig. 6) were downloaded from the Roadmap Epigenomics Project^74^. The cell type-specific enrichment was performed under the “full baseline” model^24^, which aimed to control for overlaps with annotations that are not cell type-specific. The RELI^25^ analyses were performed to identified TFs whose binding sites are enriched in the disease-associated loci. All SLE-associated SNPs and variants that are in high LD with them (*r*^2^ > 0.8) were taken as input. All the 1544 ChIP-seq datasets curated in this tool were tested in this study. The significance level and relative risk for each dataset were computed by comparing the observed intersections with expected intersections obtained from 2000 simulations.

### Identification of putative SLE genes and gene-set enrichment analysis

Putative causal gene(s) across all the SLE-associated loci outside of the HLA region were identified using DEPICT^75^. The default setting (*r*^2^ > 0.3) was used to set boundaries for each SLE associated locus. Genes within (or overlapping) the boundaries were examined and those with a *P*-value <0.05 were defined as putative causal genes. If no genes were selected at that locus, gene(s) identified from eQTL data from human whole blood^76,77^ were considered to be putatively causal. The protein-protein interaction network and the enrichment *P*-value were constructed and computed by STRING^78^ (version 11). Gene-set enrichment analysis was performed using ToppGene^27^, with the August, 2019 versions of the KEGG^79^, Reactome^80^ and mouse knockout phenotype^40^ databases. The 2017 IUIS Phenotypic Classification for Primary Immunodeficiencies^32^ (PID) was used to obtain 320 human PID genes categorized into nine phenotypic classifications.

### Trans-ancestral fine-mapping of the associated loci

The HLA region was excluded from this analysis, as extensive LD and limited genotyping of SNPs in both ancestries makes defining the best model of association difficult for this region. Disease loci with rare risk alleles (MAF < 0.01) or absent in one ancestry were also excluded, leaving 108 SLE-associated loci in the autosomes for this study. For each disease locus, all variants within the region were extracted for both ancestries. The genetic interval was determined by the closest recombination hotspots around a given disease-associated variant (defined as a recombination rate <10 cM/Mb). A fine-mapping algorithm, PAINTOR (version 3.0)^28^, was used to estimate the posterior probability of causality for each variant at a given locus based on the trans-ancestral model. For comparison, we also applied the fine-mapping algorithm on the Chinese and European SLE GWAS, separately. All analyses were run under the assumption of a single causal variant per locus, and conditional analysis was performed if multiple signals were present within a locus. The LD matrix was calculated using control samples from HK (*n* = 3324) and EUR GWAS 2 (*n* = 5379) for Chinese and European populations. Variants with a cumulative posterior probability greater than 95% were defined as putative causal variants (95% credible set).

### Identification of loci with differential effects between the two ancestries

Cochran’s Q (CQ)-test^81^ was used to examine effect-size differences between the two ancestries for all the disease-associated variants in the autosomes. If the variants were also interrogated by the Immunochip^22^ system, the association results derived from the KR, BJ, and MC cohorts were also included. CQ-test *P*-values were adjusted for a cutoff of 0.05 using the Benjamini–Hochberg method^82^. For comparison, summary association statistics on RA were downloaded from a previous study^30^ of 4873 RA cases and 17,642 controls of Asian ancestry and 14,361 RA cases and 43,923 controls of European ancestry.

### Colocalization analysis

Colocalization of association signals from the two ancestries was determined using the R package coloc^31^ on all variants with a MAF >1% and imputation (IMPUTE2^69^) INFO score >0.9 within a given disease locus. Posterior probabilities (PP) for five different configurations were evaluated at the associated loci: PP0, no association in either group; PP1, association with SLE in East Asians but not in Europeans; PP2, association with SLE in Europeans but not in East Asians; PP3, association with SLE in both ancestries but by two independent signals; PP4, association with SLE in both East Asians and Europeans by the same signal. The average PP for the five configurations were 0.24, 0.14, 0.12, 0.08, and 0.42 for the ancestry-shared loci, and 0.04, 0.67, 0.24, 0.02, and 0.03 for the ancestry-specific loci.

To control for LD differences between ancestries, SLE association signals from Chinese populations were compared with those from 27 immune-unrelated phenotypes from European populations (LD hub^20^; Supplementary Table 6) to generate baseline posterior probabilities of colocalization in the absence of a phenotypic relationship. Ancestry-shared causal effects for SLE were expected to be significantly greater than the baseline values.

### Analysis of selection signatures for the associated variants

The fixation index (*F*_st_) was used to test allele-frequency differences between the two ancestries. *F*_st_ was calculated based on the following formula^83^:

1 $$F_{\mathrm{st}} = \frac{{H_t - H_s}}{{H_t}},$$

where *H*_*t*_ is the expected proportion of heterozygosity in the pooled samples from all ethnicities based on Hardy–Weinberg equilibrium: $H_t = 2\bar p\left( {1 - \bar p} \right)$, $\bar p$ is the allele frequency in the overall pool. *H*_*s*_, the expected proportion of heterozygosity in a subpopulation (either Chinese or Europeans), is estimated as

2 $$H_s = \frac{{H_{p1} \times N_{p1} + H_{p2} \times N_{p2}}}{{N_{p1} + N_{p2}}},$$

where *H*_*pi*_ is the expected heterozygosity in the *i*^th^ subpopulation estimated by the allele frequency in that subpopulation under Hardy-Weinberg equilibrium. *N*_*pi*_ is the sample size of the *i*^th^ subpopulation.

Potential selective sweeps in respective ancestries were examined using the Integrated Haplotype Score (iHS) method, which measures the extended haplotype homozygosity for the ancestral allele relative to the derived allele^84^. Raw iHS scores were computed using the R package rehh^85^ and normalized by different frequency bins (50 bins over the range 0 to 1). Large negative standardized iHS values indicate long haplotypes carrying the derived allele, while large positive values suggest long haplotypes with the ancestral allele. The *F*_st_ and iHS values analyzed in this study were estimated using control subjects from HK (*n* = 3324) and EUR GWAS 2 (*n* = 5379). Standardized iHS scores based on the 1000 Genomes Project were downloaded from a previous study^45^ for comparison.

### Calculation of polygenic risk scores

Polygenic risk scores (PRS) for individuals were computed using lassosum^48^, a penalized regression framework. The meta-analysis results on Europeans were used to calculate PRS for individuals of Chinese ancestry, and vice versa. LD information among SNPs was calculated from the testing dataset. These analyses were repeated using LDpred^49^.

The GZ SLE GWAS cohort was used as a test dataset to evaluate the influence of training data from different ancestries. Two predictors were constructed using lassosum based on meta-analysis results from: (1) HK and CC GWAS, 2618 cases and 7446 controls; (2) European GWAS, 4576 cases and 8039 controls. To control for influence from different sample sizes, 1500 cases and 1500 controls were randomly chosen from the Chinese and European populations to train two same-size predictors (repeated 3 times). PRS values generated from each test were scaled to a mean of 0 and a standard deviation of 1, and then evaluated based on the area under the ROC curve (AUC). The values and the 95% confidence intervals were calculated using the R package pROC^86^ and the optimal cut-off, the point that maximizes the sum of sensitivity and specificity, for case-control classification was estimated using the coords function.

### Reporting summary

Further information on research design is available in the Nature Research Reporting Summary linked to this article.

## Supplementary information

Supplementary Information

Reporting Summary

Description of Additional Supplementary Files

Supplementary Data 1

Supplementary Data 2

Supplementary Data 3

Supplementary Data 4

Supplementary Data 5

## Data Availability

Genome-wide association summary statistics for the East Asian populations can be accessed through the GWAS Catalog (GCST90011866). The data for the European populations are available at http://insidegen.com/ and http://urr.cat/data/GWAS_SLE_summaryStats.zip. The ImmunoChip data are publicly available for download at https://www.ncbi.nlm.nih.gov/pmc/articles/PMC4767573/bin/NIHMS747721-supplement-3.xlsx. Summary association statistics for other phenotypes are downloaded from LD hub (http://ldsc.broadinstitute.org/). Summary statistics for eQTL results are retrieved from Blood eQTL browser (https://genenetwork.nl/bloodeqtlbrowser/). Protein–protein interaction information is downloaded from STRING database (https://string-db.org/). Histone modifications across cell types are downloaded from the Roadmap Epigenomics Project (http://www.roadmapepigenomics.org/).

## Code availability

The code that support the findings of this study are available from the corresponding author upon request.
