# Supplementary material for: Identification of 38 novel loci for systemic lupus erythematosus and genetic heterogeneity between ancestral groups
Source: Nat Commun. 2021 Feb 3;12:772. doi: 10.1038/s41467-021-21049-y (PMC7858632; doi:10.1038/s41467-021-21049-y)
Supplement: Supplementary file 1 — Supplementary Information [file 41467_2021_21049_MOESM1_ESM.pdf]

Supplementary Figures and Tables

Supplementary Fig. 1 Principal component (PC) analysis for individuals from Hong Kong, Guangzhou and Central China GWAS. 4

Supplementary Fig. 2 PC analysis for individuals from the Chinese and European GWAS. 5

Supplementary Fig. 3 Manhattan plots for the SLE GWAS from Chinese populations. 6

Supplementary Fig. 4 Manhattan plots for the SLE GWAS from European populations. 8

Supplementary Fig. 5 Enrichment of SLE heritability across 28 core annotations (not specific for cell-type). 9

Supplementary Fig. 6 Enrichment of SLE heritability across different cell types based on H3K4me1 and H3K4me3 modifications. 10

Supplementary Fig. 7 Protein-Protein interaction (PPI) network corresponding to putative disease genes across SLE susceptibility loci. 11

Supplementary Fig. 8 Trans-ancestral fine-mapping results at the *TNFSF4* and *WDFY4* loci. 12

Supplementary Fig. 9 Forest plots for the disease-associated loci with heterogeneity between East Asian (EAS) and European (EUR) populations. 13

Supplementary Fig. 10 Regional plots for the loci showing significant differences in effect-size estimates between the two ancestral groups. 15

Supplementary Fig. 11 Colocalization of East Asian (EAS) SLE association signals with European SLE associations and other non-immune system related diseases. .. 16

Supplementary Fig. 12 Comparison of risk allele frequency and standardized iHS scores between East Asians and Europeans for SLE-associated variants. 17

Supplementary Fig. 13 Disease risk prediction accuracy based on polygenetic risk scores (PRS) between the two ancestral group populations. 18

Supplementary Fig. 14 Performance of PRS for GZ samples based on data from Chinese and European populations with equivalent sample size. 19

Supplementary Table 1 Evaluation of genotyping accuracy between different BeadChips 20

Supplementary Table 2 Summary of SLE cohorts from East Asian (EAS) and European (EUR) populations 21

Supplementary Table 3 Pathway enrichment analysis based on putative SLE genes 22

Supplementary Table 4 Groups of putative SLE genes based on heterogeneity in effect-size estimates 24

Supplementary Table 5 SLE-associated loci with heterogenity between East Asian and European populations 25

Supplementary Table 6 Twenty-seven immune-unrelated phenotypes studied in European populations used to compare with East Asian SLE association signals in colocalization analyses 26

a

HK GWAS

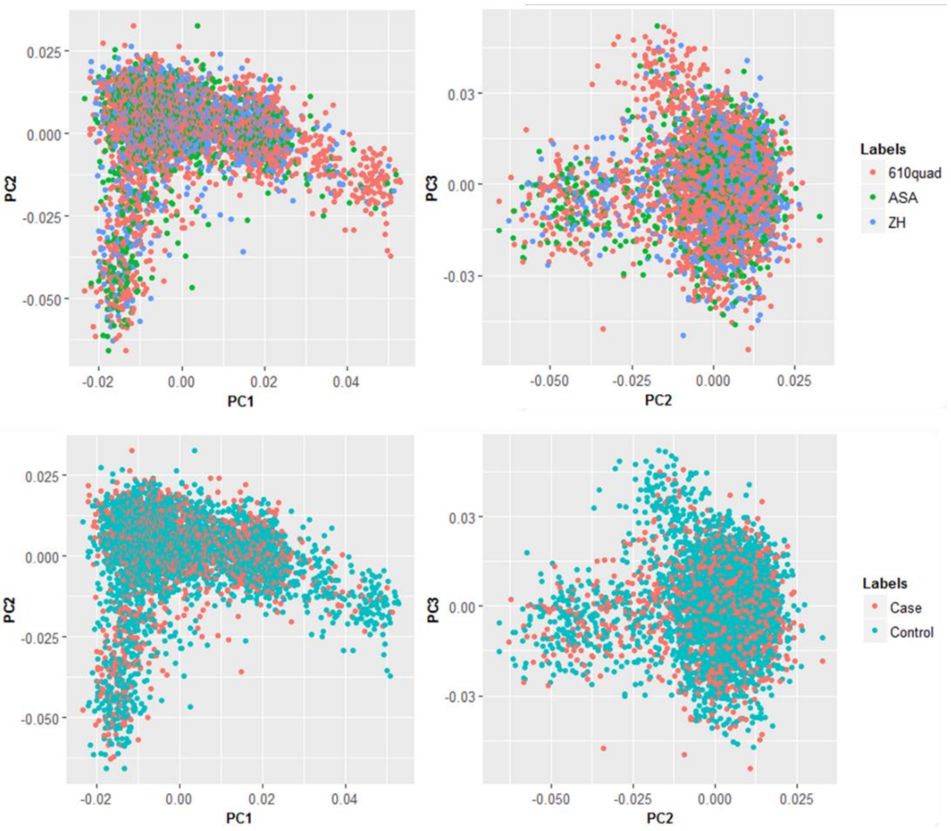

b

GZ GWAS

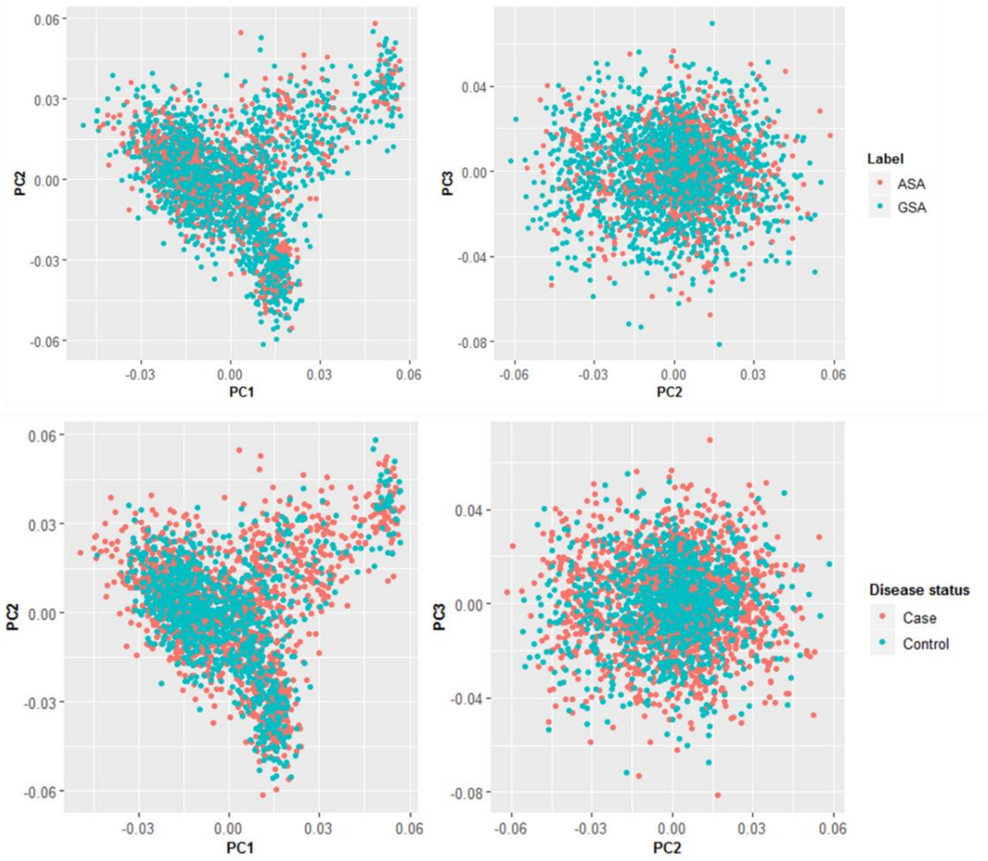

c

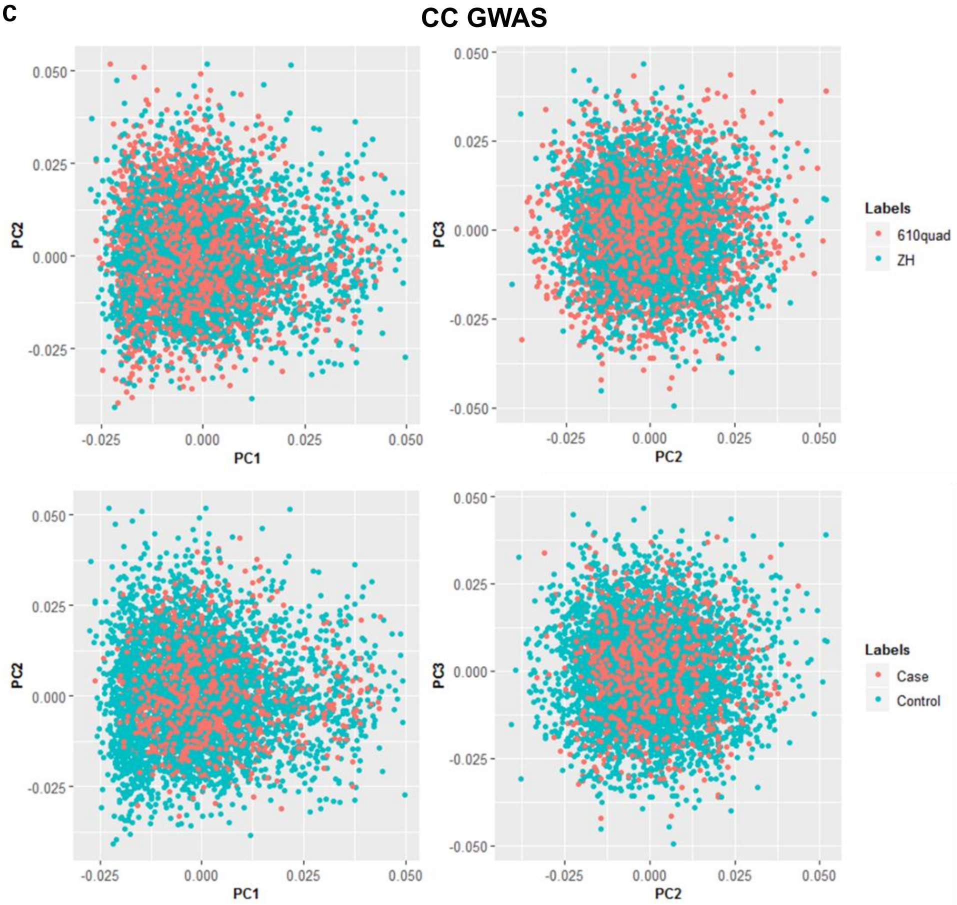

Supplementary Fig. 1 Principal component (PC) analysis for individuals from Hong Kong (HK, **a**), Guangzhou (GZ, **b**) and Central China (CC, **c**) GWAS.

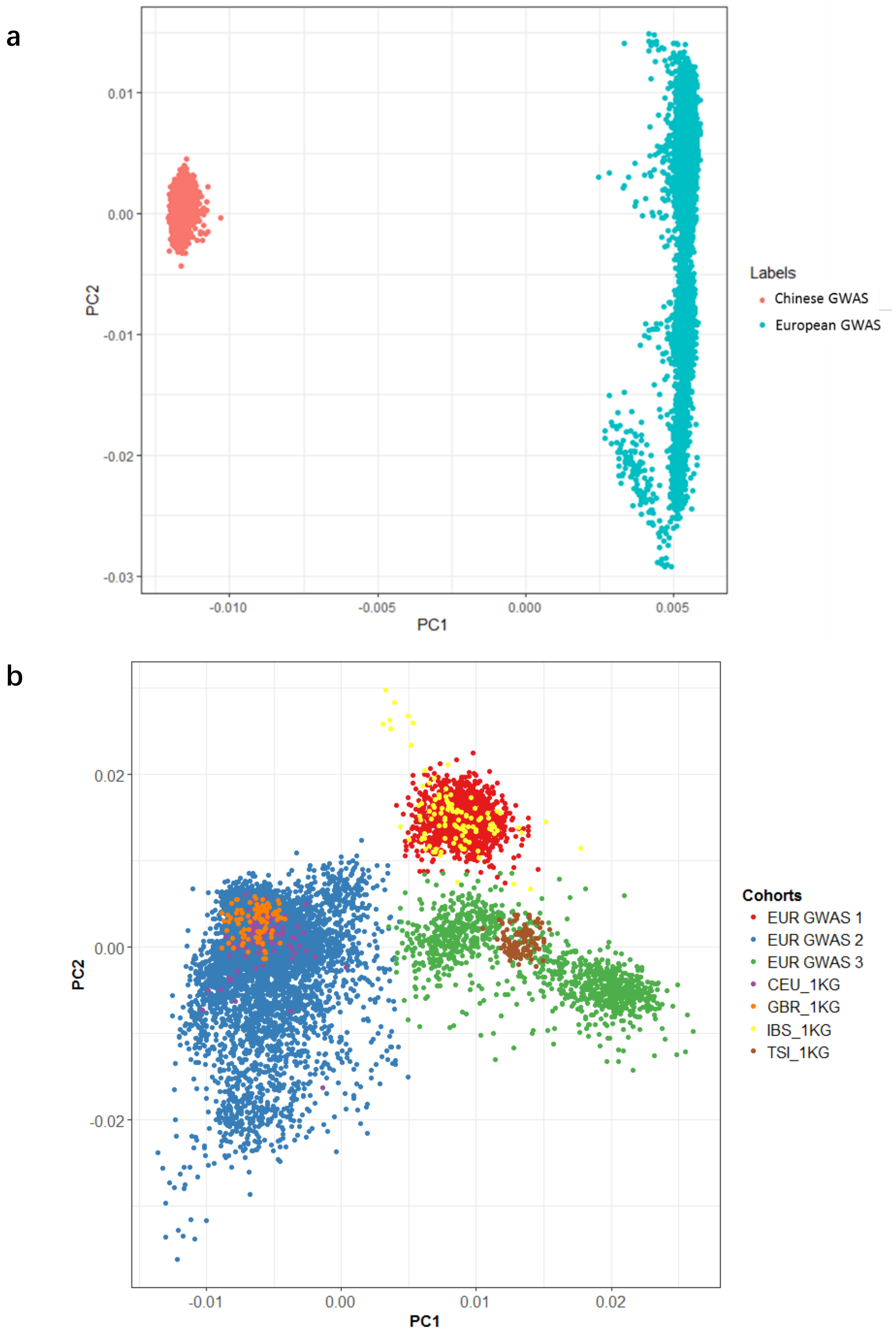

Supplementary Fig. 2 PC analysis for individuals from the Chinese and European GWAS. **a**, comparison of PC for individuals of Chinese and European ancestries used in this study. **b**, Comparing the PCs for individuals in European GWAS with subjects from the 1,000 Genomes Project. Samples in EUR GWAS 1 overlapped individuals from Iberian population of Spain in the 1000 Genomes Project (IBS_1KG). Samples in EUR GWAS 2 overlapped individuals of northern and western European Ancestry (CEU_1KG and GBR_1KG). Samples in EUR GWAS 3 overlapped individuals from Toscani in Italy (ITS_1KG).

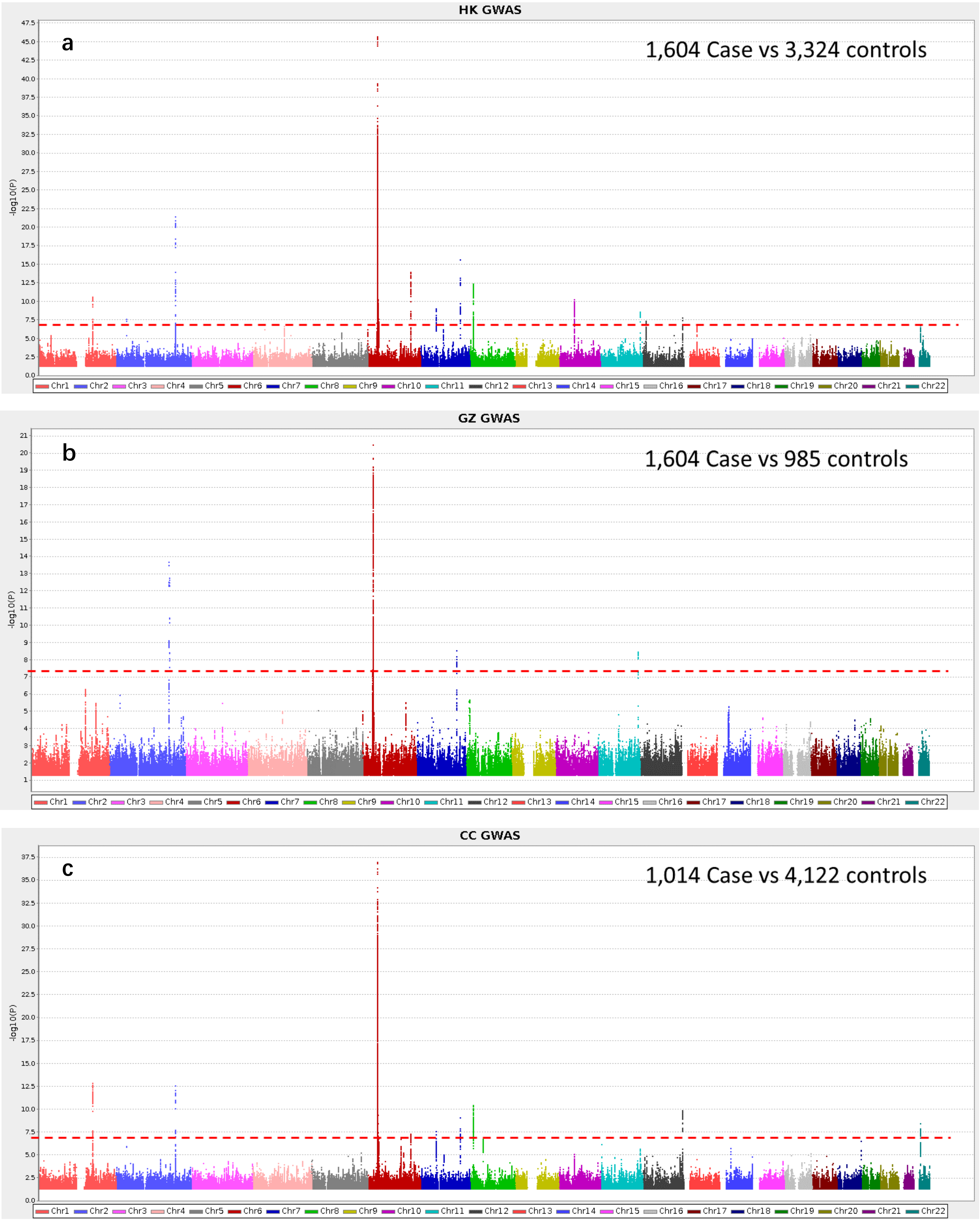

Supplementary Fig. 3 Manhattan plots for the SLE GWAS from Chinese populations.

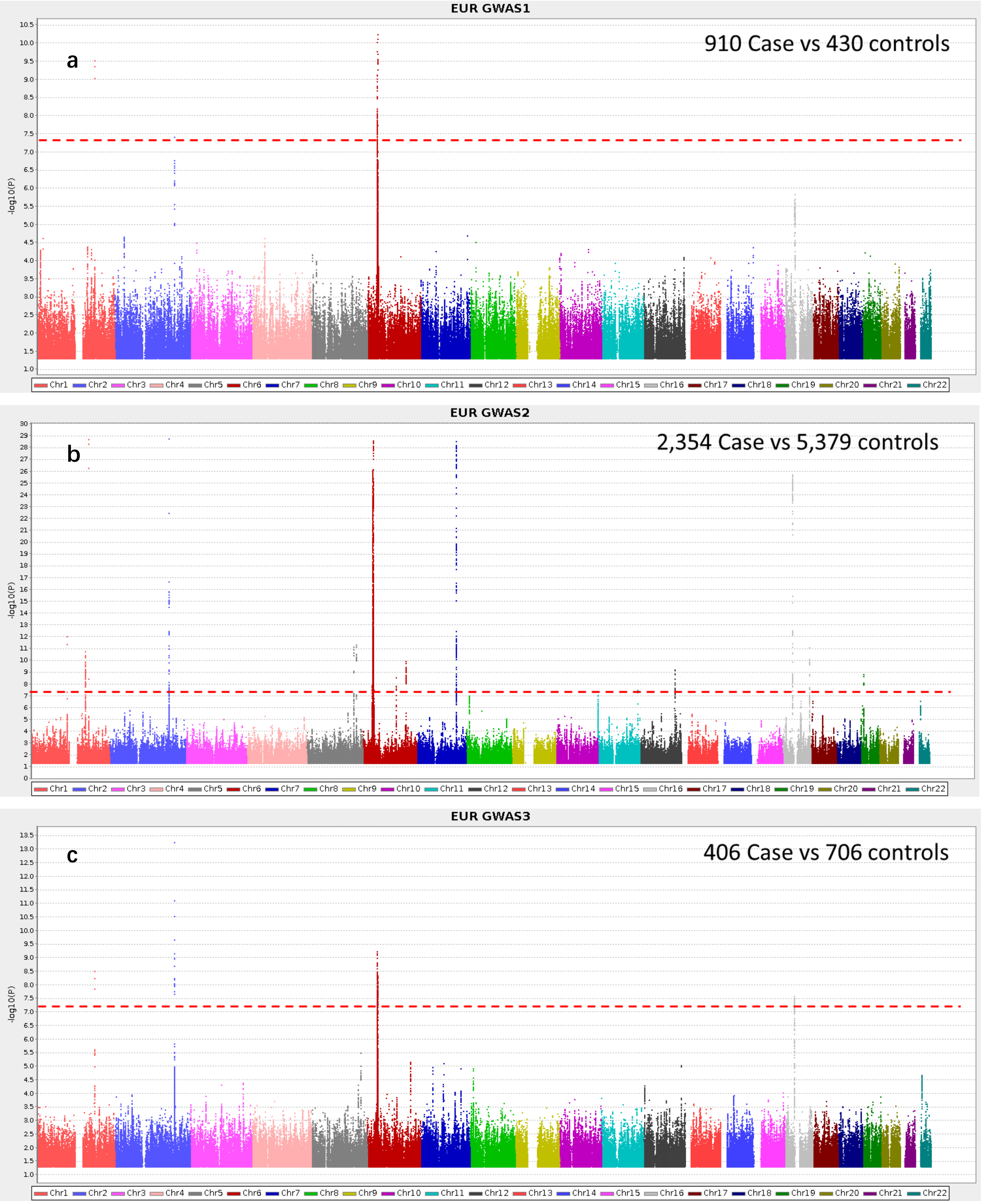
EUR GWAS1
a
910 Case vs 430 controls
-log10(P)
Chr1 Chr2 Chr3 Chr4 Chr5 Chr6 Chr7 Chr8 Chr9 Chr10 Chr11 Chr12 Chr13 Chr14 Chr15 Chr16 Chr17 Chr18 Chr19 Chr20 Chr21 Chr22
EUR GWAS2
b
2,354 Case vs 5,379 controls
-log10(P)
Chr1 Chr2 Chr3 Chr4 Chr5 Chr6 Chr7 Chr8 Chr9 Chr10 Chr11 Chr12 Chr13 Chr14 Chr15 Chr16 Chr17 Chr18 Chr19 Chr20 Chr21 Chr22
EUR GWAS3
c
406 Case vs 706 controls
-log10(P)
Chr1 Chr2 Chr3 Chr4 Chr5 Chr6 Chr7 Chr8 Chr9 Chr10 Chr11 Chr12 Chr13 Chr14 Chr15 Chr16 Chr17 Chr18 Chr19 Chr20 Chr21 Chr22

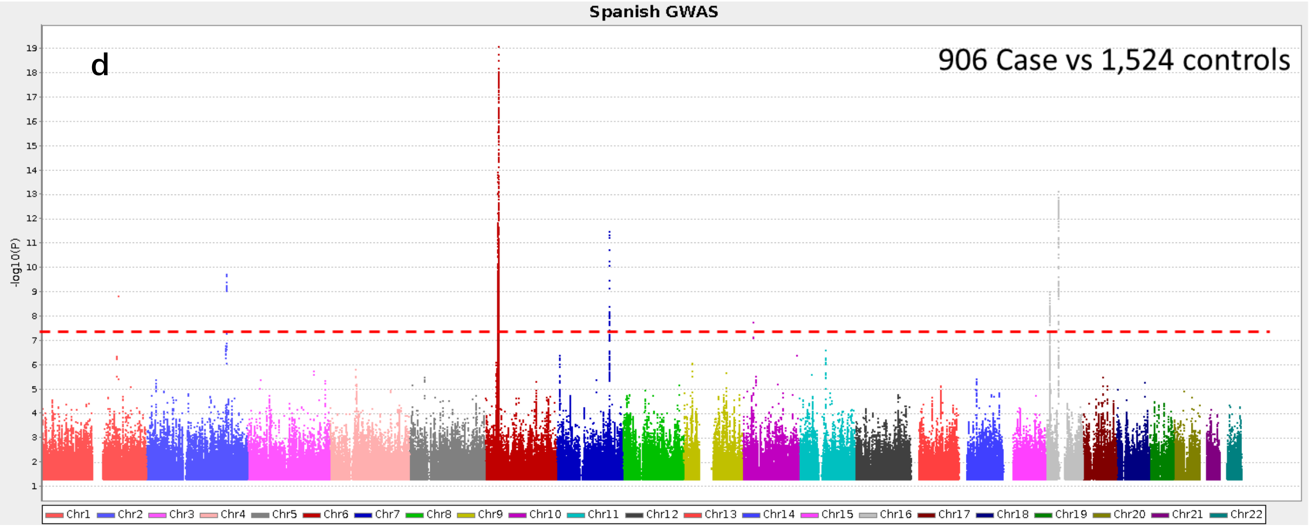

Supplementary Fig. 4 Manhattan plots for the SLE GWAS from European populations.

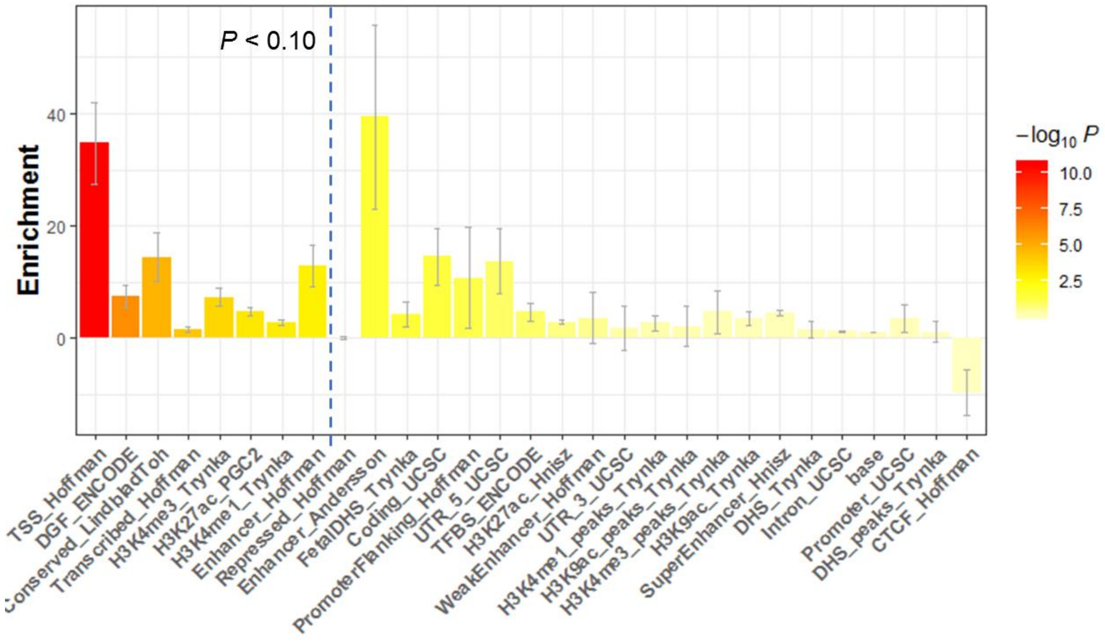

Supplementary Fig. 5 Enrichment of SLE heritability across 28 core annotations (not specific for cell-type). Error bars represent jackknifed standard errors around the estimates of enrichment based on LD score regression (two-sided). This analysis was performed by using the trans-ancestral GWAS meta-analysis result involving a total of 8,798 independent SLE cases and 16,470 controls (Methods).

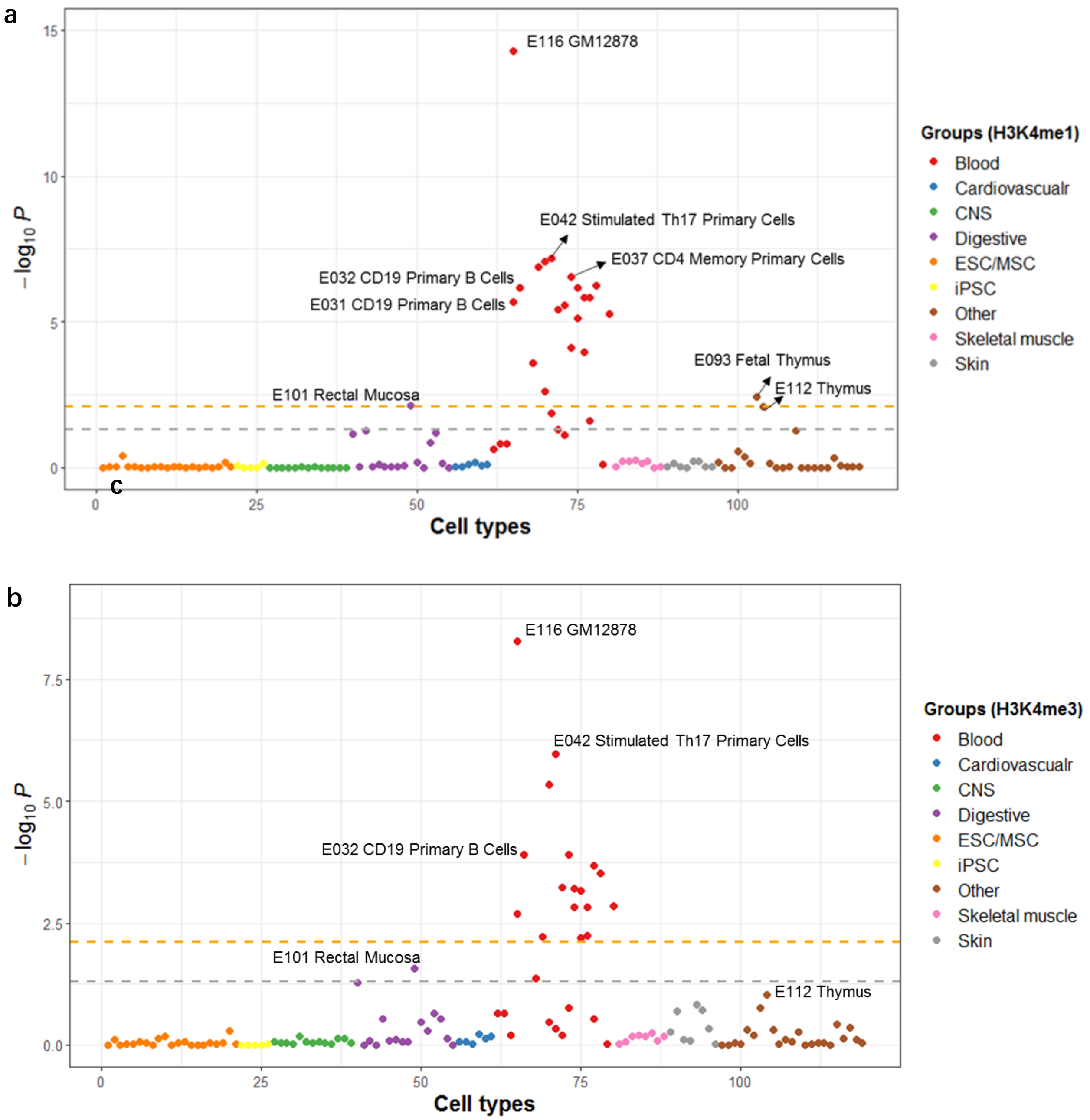

Supplementary Fig. 6 Enrichment of SLE heritability across different cell types based on H3K4me1 **(a)** and H3K4me3 modifications **(b)**. Epigenomic data were obtained from the Roadmap Epigenomics Project and categorized into nine tissue/organ groups: blood, cardiovascular, gastrointestinal, central nervous system (CNS), skeletal muscle, skin, embryonic (ESC) or mesenchymal (MSC) stem cells, induced pluripotent stem cells (iPS) and others. The orange lines indicate a FDR threshold of 0.05 and the gray lines indicate a two-sided *P*-value threshold 0.05.

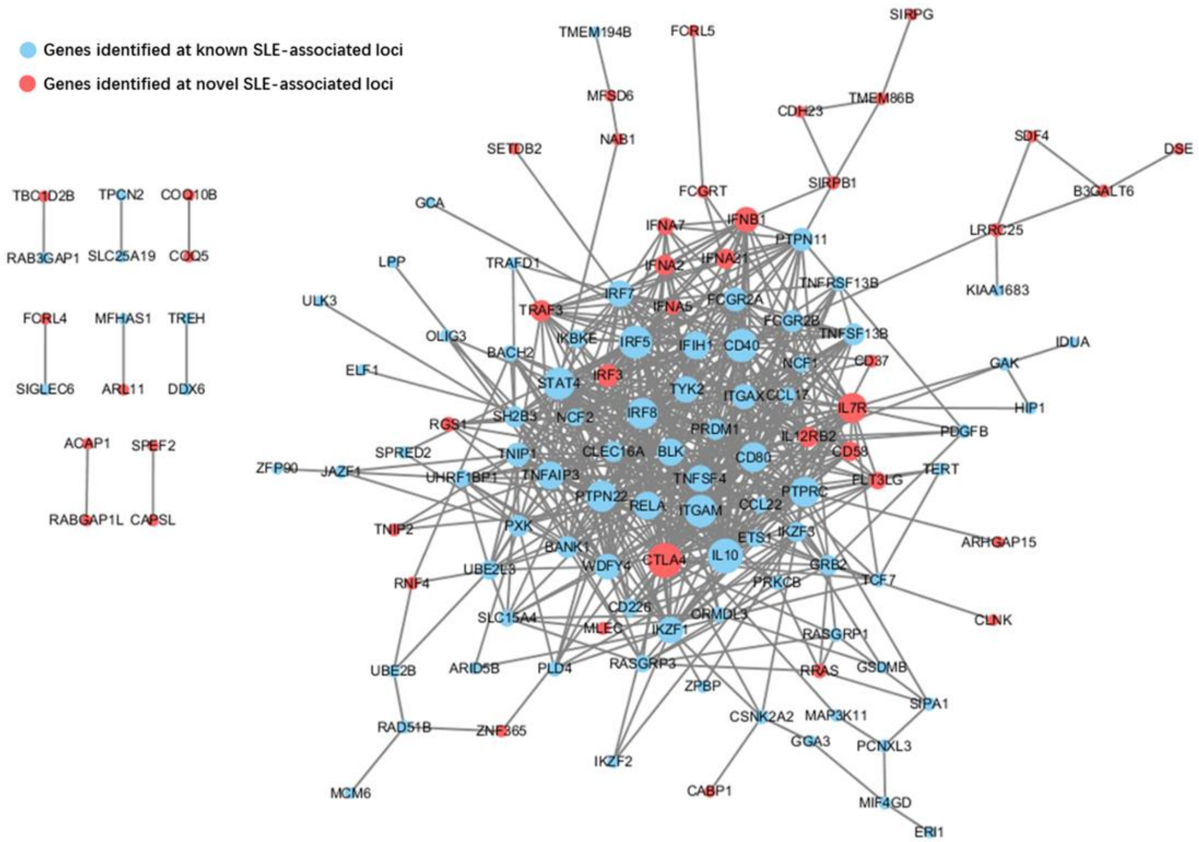

Supplementary Fig. 7 Protein-Protein interaction (PPI) network corresponding to putative disease genes across SLE susceptibility loci. Genes identified at the novel SLE-associated loci are labeled in red, and genes found at the known SLE-associated loci are labeled in blue. The PPI information was retrieved from the STRING database (version 11.0).

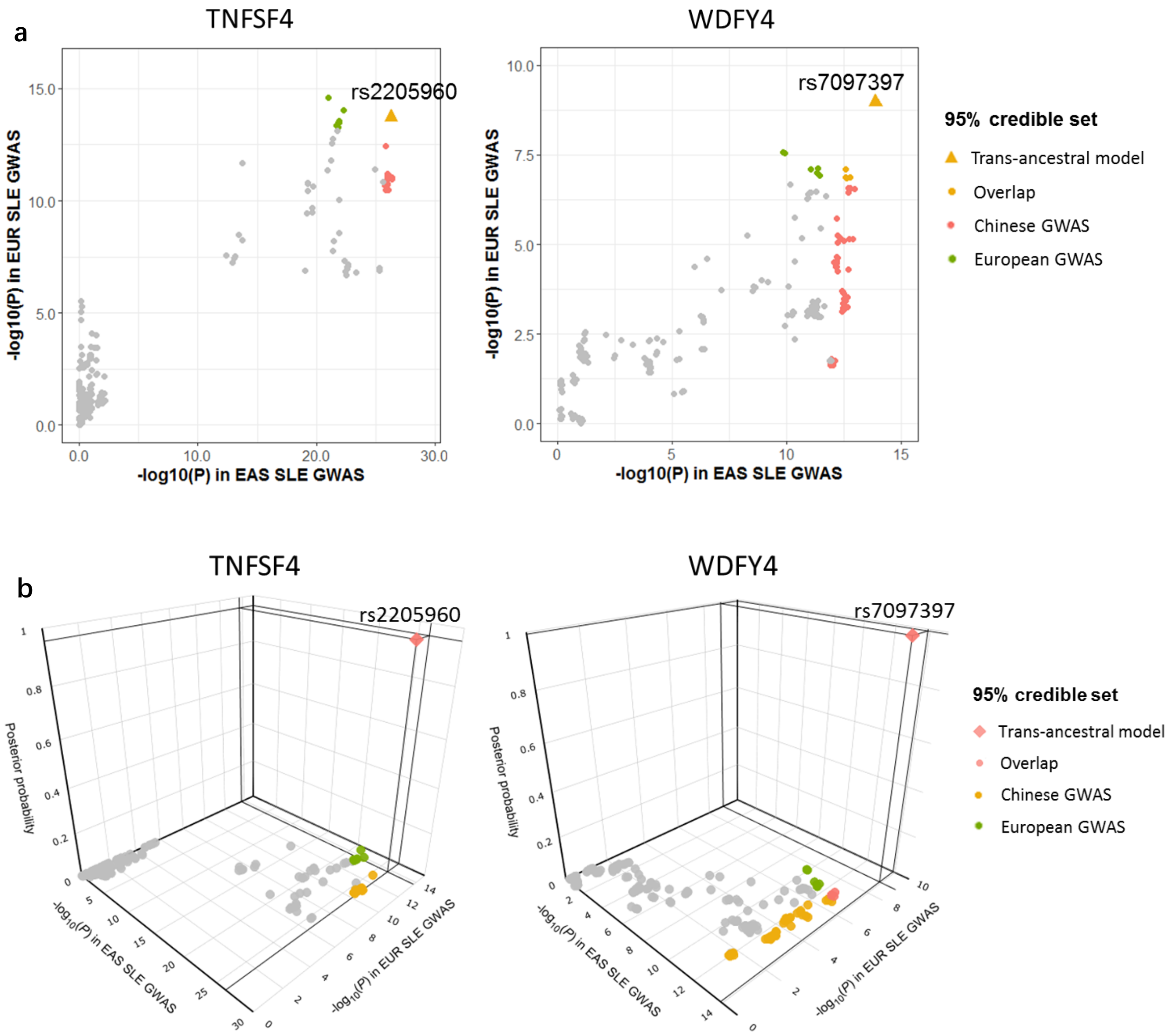

Supplementary Fig. 8 Trans-ancestral fine-mapping results at the *TNFSF4* and *WDFY4* loci. **a,** X-axis indicates the log10 transformed association signal in the Chinese SLE GWAS, and Y-axis indicates the log10 transformed association signal in the European SLE GWAS. SNPs included in the 95% credible sets that were identified using the Chinese and European GWAS alone are labeled in red and green, respectively and yellow dots indicate variants found in both ancestral groups. The variant within 95% credible set that was identified using trans-ancestral fine-mapping model is labeled as a triangle. **b,** As in **a**, with the additional Z-axis showing the posterior probability of causality for each variant based on trans-ancestral fine-mapping model. For the analysis of the *TNFSF4* locus, the 95% credible set was reduced from 9 variants using the European GWAS alone to a single variant after the trans-ancestral analysis. The posterior probability of causality for the top variant, rs2205960, increased from 0.07 to 0.96, owing to the differential LD with other variants between the two ancestral groups. Similarly, a unique putative variant was also identified at the *WDFY4* locus (**Supplementary Data 5**).

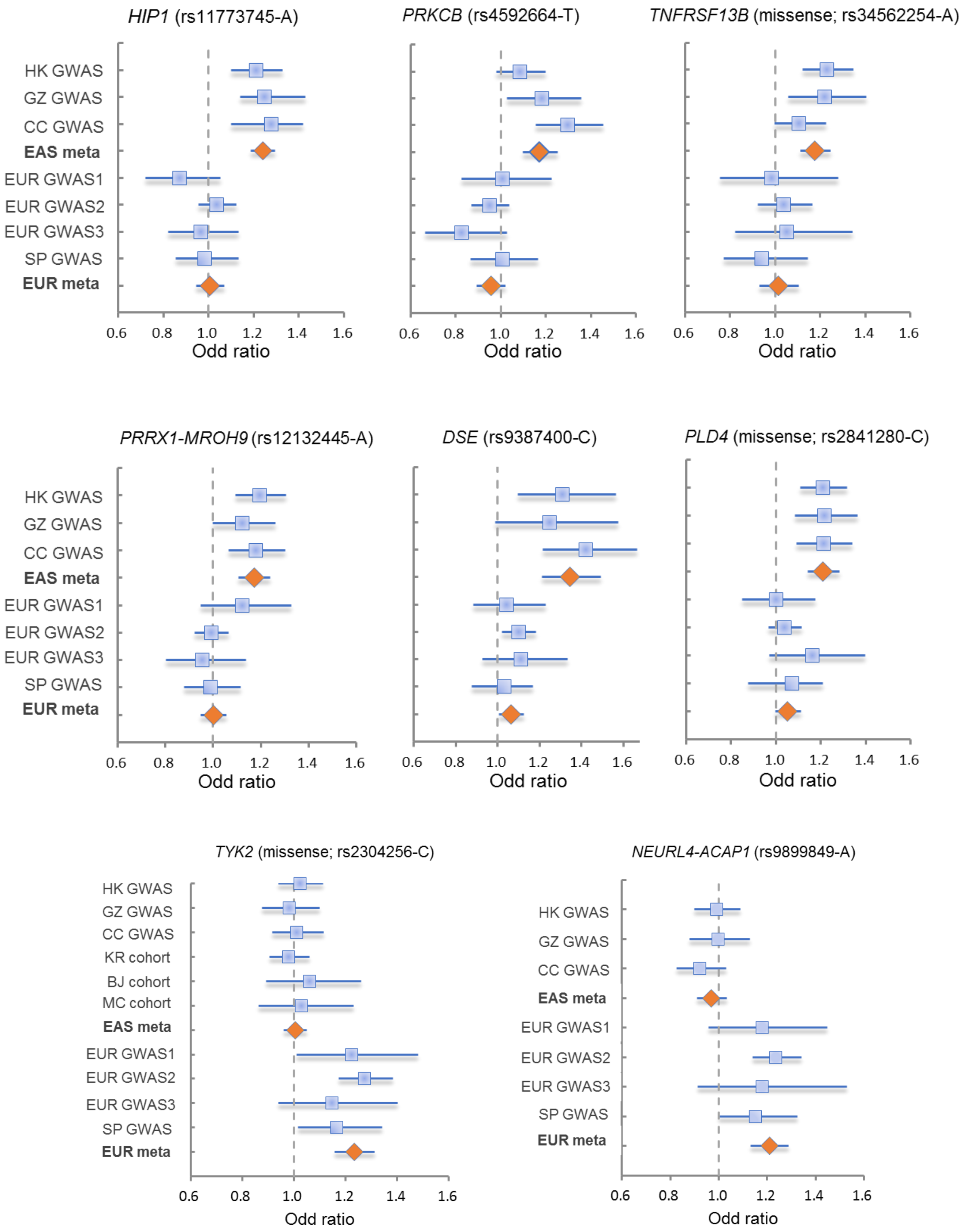

Supplementary Fig. 9 Forest plots for the disease-associated loci with heterogeneity between East Asian (EAS) and European (EUR) populations. Standard error bars of odds ratio (OR) represent 95% confidence intervals of the estimates and the central dots represent OR in different cohorts.

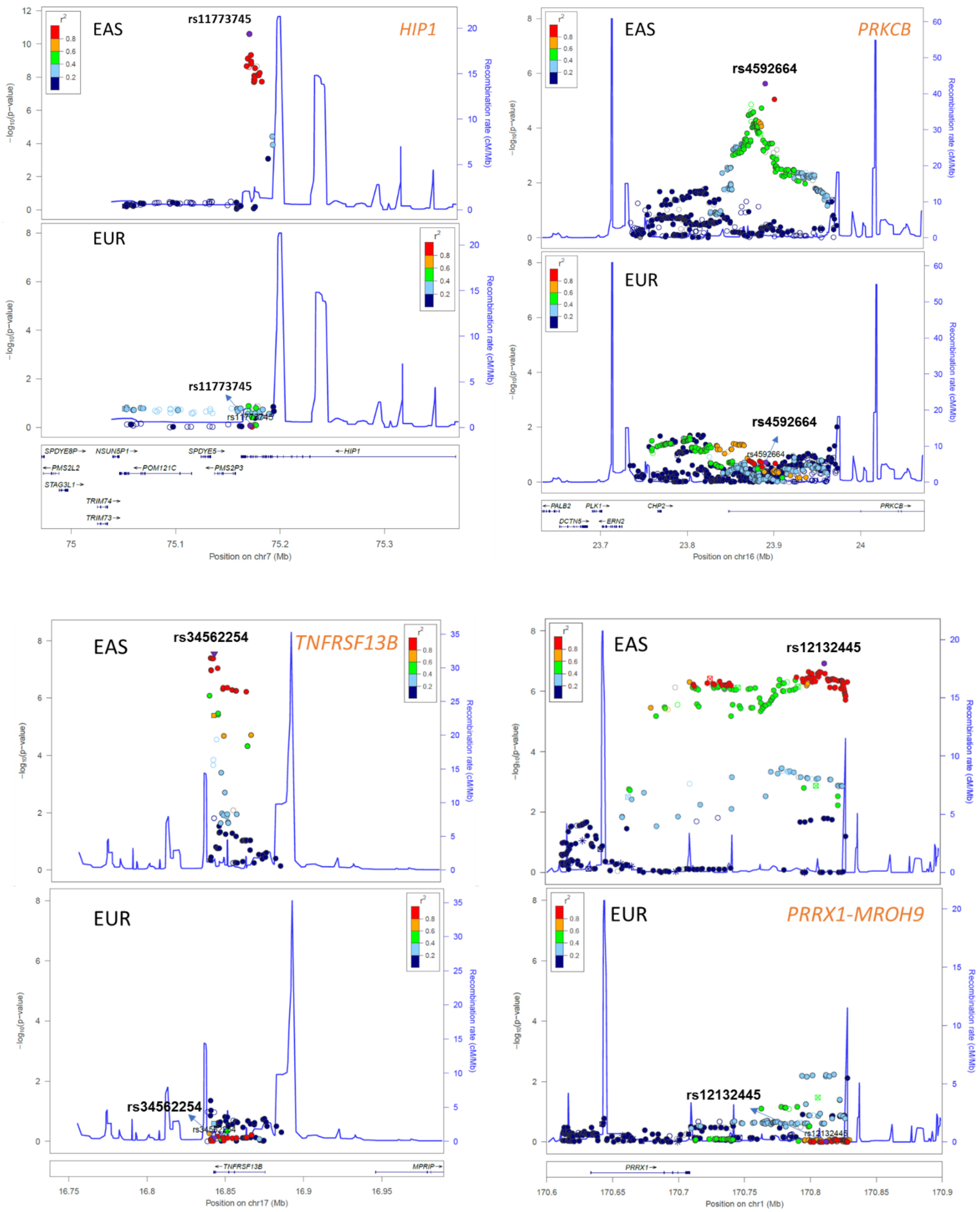
EAS
EUR
rs11773745
HIP1
Position on chr7 (Mb)
SPDYE8P
NSUN5P1
SPDYE5
PMS2L2
POM121C
PMS2P3
STAG3L1
TRIM74
TRIM73
rs4592664
PRKCB
Position on chr16 (Mb)
PALB2
PLK1
CHP2
DCTN5
ERN2
rs34562254
TNFRSF13B
MPRIP
Position on chr17 (Mb)
rs12132445
PRRX1-MROH9
PRRX1
Position on chr1 (Mb)
Recombination rate (cM/Mb)

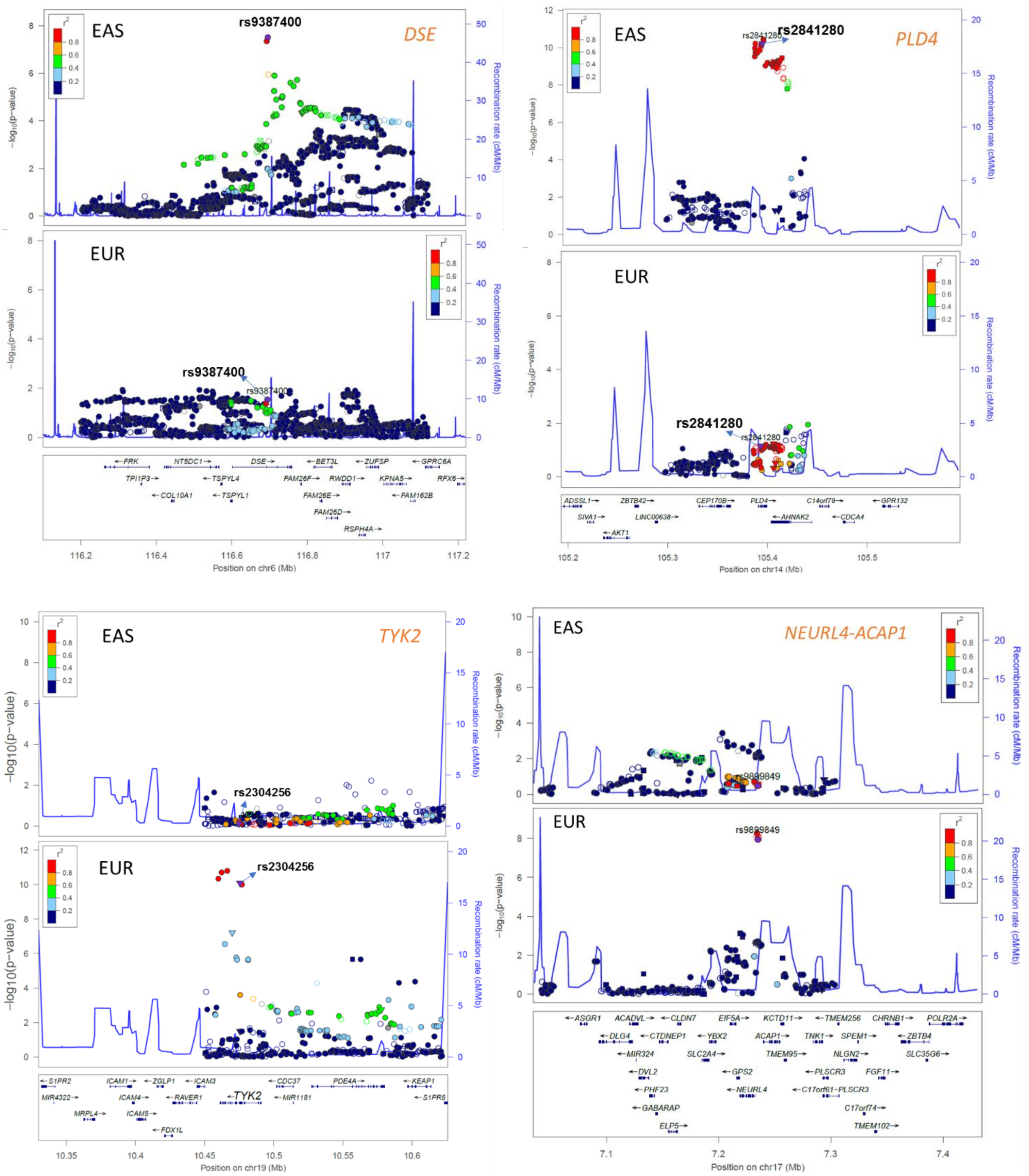

Supplementary Fig. 10 Regional plots for the loci showing significant differences in effect-size estimates between the two ancestral groups.

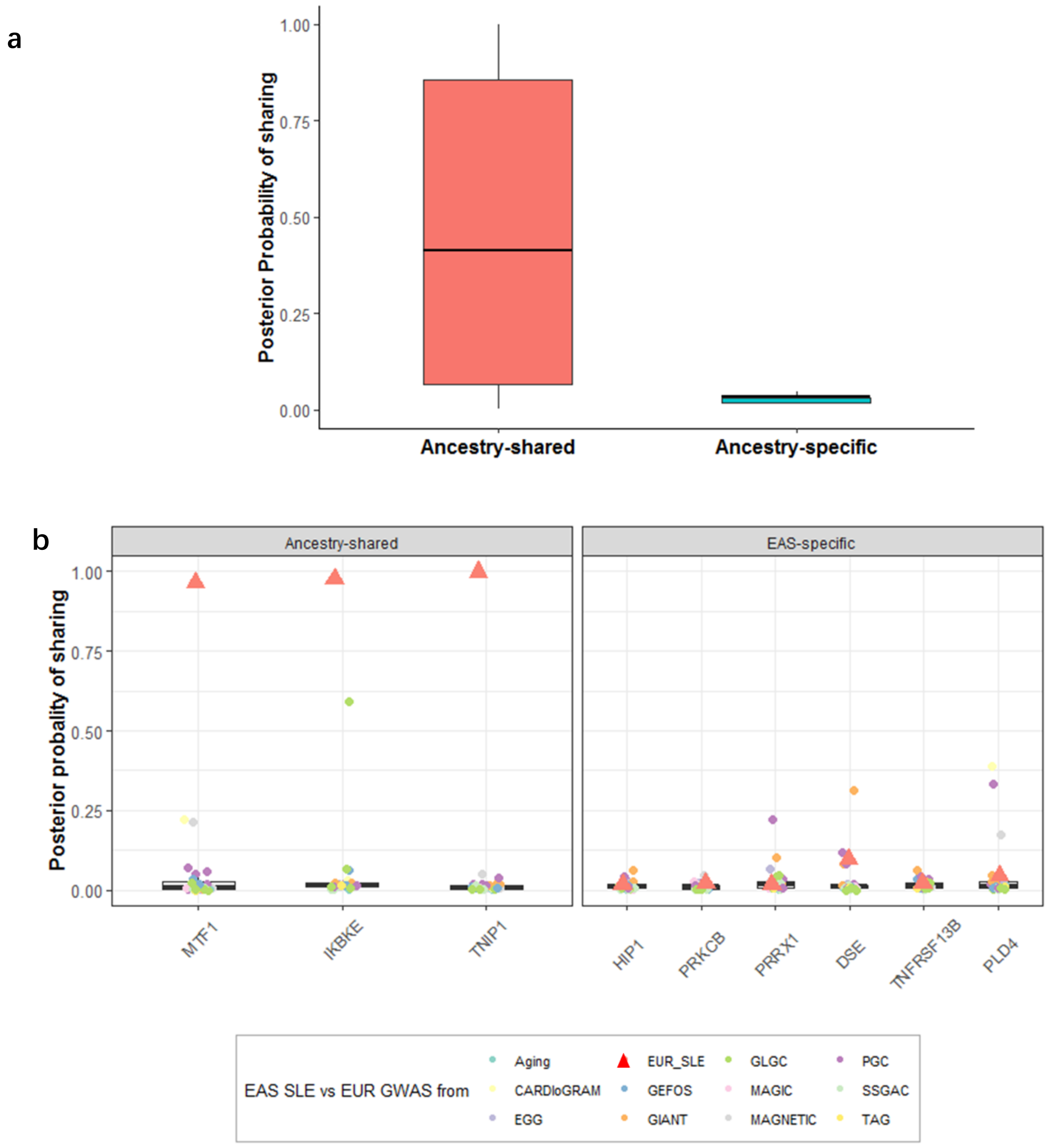

Supplementary Fig. 11 Colocalization of East Asian (EAS) SLE association signals with European SLE associations and other non-immune system related diseases. **a**, Posterior probability of sharing for each ancestry-shared (n = 79 independently associated loci; left) and ancestry-specific SLE loci (n = 8 independently associated loci; right). The upper and lower bounds of box represent the first quartile (Q1 or 25th percentile) and the third quartile (Q3 or 75th percentile) and the central line indicates the median. The two lines outside the box extend to the highest and lowest observations. **b**, Red triangles represent the posterior probability of SLE association signals between East Asian and European ancestries. Other symbols represent the colocalization of Chinese SLE association signals with those in European populations for 27 phenotypes unrelated to immunity. Summary statistics for the 27 phenotypes were downloaded from the results of 11 consortium studies: Aging, Global Lipids Genetics Consortium (GLGC), Psychiatric Genomics Consortium (PGC), Coronary Artery Disease Genome-wide Replication and Meta-analysis Consortium (CARDIoGRAM), Genetic Factors for Osteoporosis Consortium (GEFOS), Meta-analyses of Glucose and Insulin-related traits Consortium (MAGIC), Social Science Genetic Association Consortium (SSGAC), Early Growth Genetics (EGG), Genetic Investigation of Anthropometric Traits (GIANT), Myocardial Applied Genomics Network consortium (MAGNET) and Tobacco and Genetics Consortium (TAG).

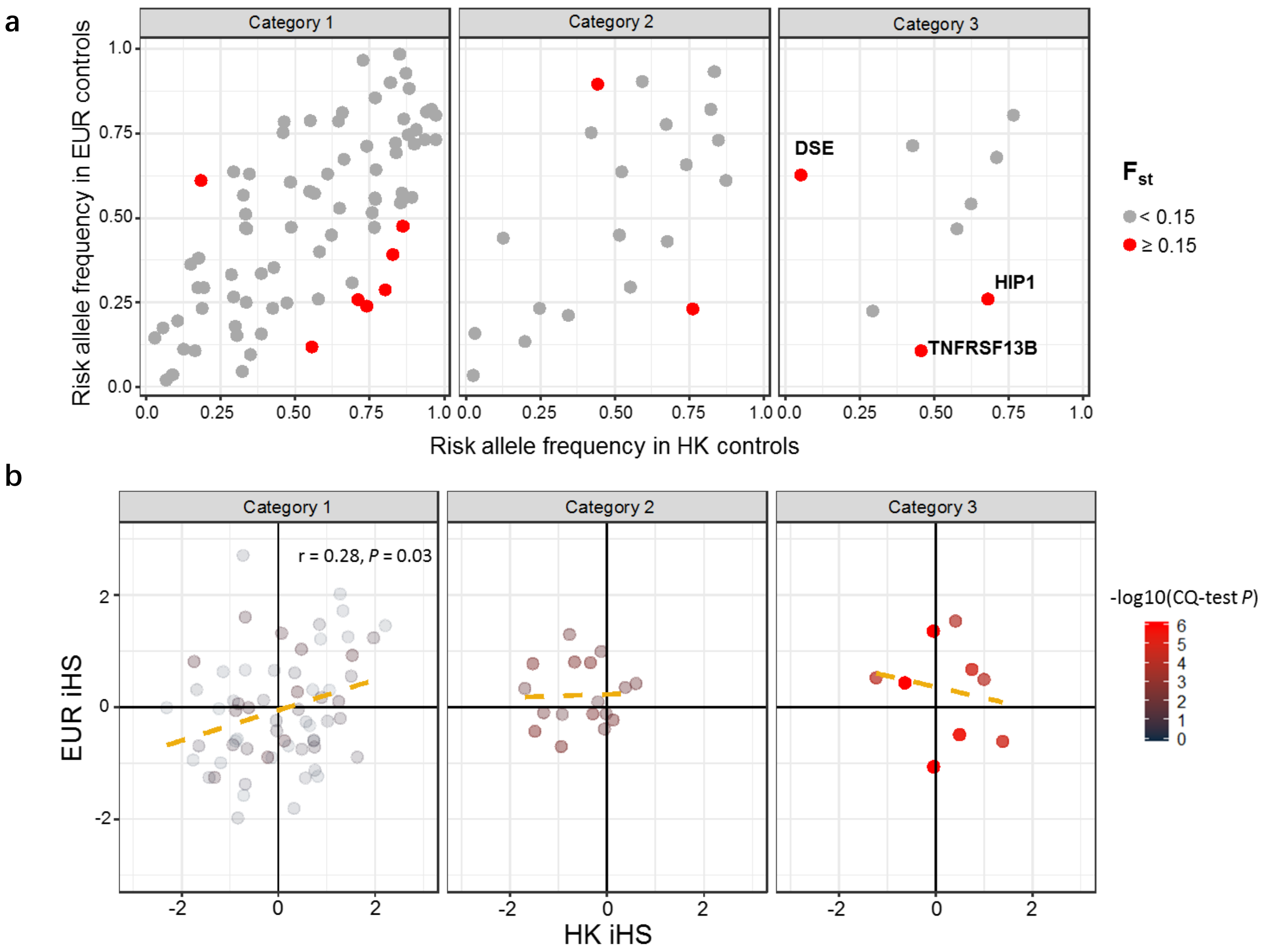

Supplementary Fig. 12 Comparison of risk allele frequency and standardized iHS scores between East Asians and Europeans for SLE-associated variants. **a**. X-axis is the SLE risk allele frequency in a Hong Kong population (n = 3,324), and the y-axis is the risk allele frequency in EUR GWAS 2 (n = 5,379). The disease variants showing significant frequency variation (F_{st} > 0.15) between ethnicities are shown in red. **b**. X-axis is the standardized iHS score estimated using data from a Hong Kong population (n = 3,324), y-axis is the score estimated using data from the European populations (n = 5,379).

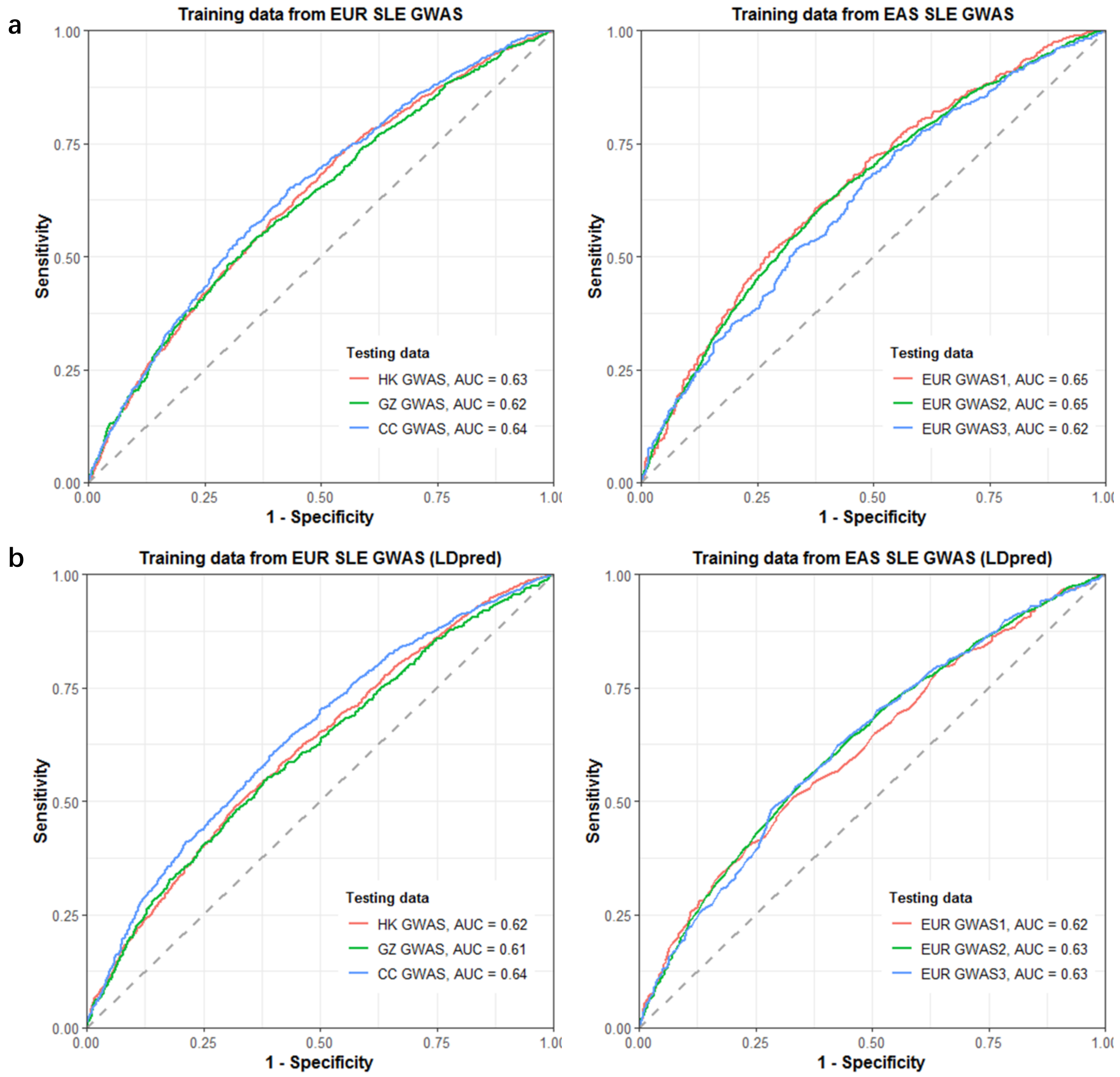

Supplementary Fig. 13 Disease risk prediction accuracy based on polygenetic risk scores (PRS) between the two ancestral group populations. SLE PRS for individuals in the East Asian cohorts were calculated based on the summary statistics from the European GWAS (left panel), and vice versa (right panel). Data on a total of 4,222 SLE cases and 8,431 controls was included in the summary statistics of Chinese SLE GWAS, and data on a total of 4,576 cases and 8,039 controls were included in the summary statistics of European SLE GWAS. **a**. Performance of PRS calculated by lassosum for individuals from different cohorts; **b**. Performance of PRS calculated by LDpred, another algorithm for PRS estimation.

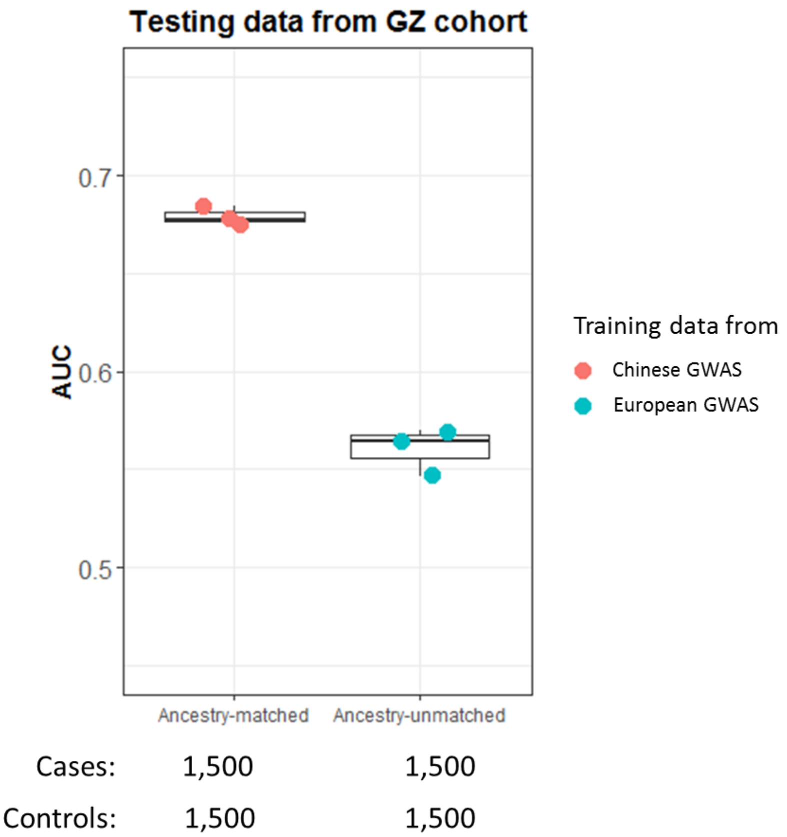

Supplementary Fig. 14 Performance of PRS for GZ samples based on data from Chinese and European populations with equivalent sample size. To control for the influence of sample size 1,500 cases and 1,500 controls were randomly selected to train the predictors for the populations (red, Chinese; blue, European). These procedures were repeated 3 times, and the performance measured by AUC for each test is represented by the dot.

Supplementary Table 1 Evaluation of genotyping accuracy between different BeadChips

Sample	BeadChips	#discordant SNPs	#overlapped SNPs	%concordant rate
1	610-Quad vs ZhongHua	11	293103	100.00%
2	610-Quad vs ZhongHua	10	293113	100.00%
3	610-Quad vs ZhongHua	35	292529	99.99%
4	610-Quad vs ZhongHua	10	292855	100.00%
5	610-Quad vs ZhongHua	5	293078	100.00%
6	610-Quad vs ZhongHua	28	292571	99.99%
7	610-Quad vs ZhongHua	4	293110	100.00%
8	610-Quad vs ZhongHua	10	293119	100.00%
9	GSA vs ASA	1	145804	100.00%
10	GSA vs ASA	6	145722	100.00%
11	GSA vs ASA	9	145626	99.99%
12	GSA vs ASA	8	145542	99.99%
13	GSA vs ASA	7	145616	100.00%
14	GSA vs ASA	5	145537	100.00%

Supplementary Table 2 Summary of SLE cohorts from East Asian (EAS) and European (EUR) populations

Cohorts	Ancestry	#Case	#Controls	#Total
HK GWAS	EAS	1,604	3,324	4,928
GZ GWAS	EAS	1,604	985	2,589
CC GWAS	EAS	1,014	4,122	5,136
KR cohort	EAS	1,710	6,836	8,546
BJ cohort	EAS	490	493	983
MC cohort	EAS	285	287	572
EUR GWAS1	EUR	910	430	1,340
EUR GWAS2	EUR	2,354	5,379	7,733
EUR GWAS3	EUR	406	706	1,112
SP GWAS	EUR	906	1524	2,430
**Total**		**11,283**	**24,086**	**35,369**

HK: Hong Kong; GZ: Guangzhou; CC: Central China; KR: Korea; BJ: Beijing; MC: Chinese in Malaysia; SP: Spain

Supplementary Table 3 Pathway enrichment analysis based on putative SLE genes

Name	Source	*P*-value	FDR
Cytokine Signaling in Immune system	REACTOME	3.05E-12	2.18E-08
Interferon alpha/beta signaling	REACTOME	2.23E-11	7.98E-08
Toll-like receptor signaling pathway	KEGG	1.34E-10	2.91E-07
Measles	KEGG	1.98E-10	2.91E-07
RIG-I/MDA5 mediated induction of IFN-alpha/beta pathways	REACTOME	2.03E-10	2.91E-07
RIG-I-like receptor signaling pathway	KEGG	5.83E-10	6.54E-07
TRAF6 mediated IRF7 activation	REACTOME	6.41E-10	6.54E-07
Regulation of IFNA signaling	REACTOME	2.25E-09	1.80E-06
Cytokine-cytokine receptor interaction	KEGG	2.27E-09	1.80E-06
Hepatitis B	KEGG	5.93E-09	4.24E-06
Herpes simplex infection	KEGG	1.06E-08	6.91E-06
Jak-STAT signaling pathway	KEGG	1.47E-08	8.76E-06
Autoimmune thyroid disease	KEGG	2.11E-08	1.16E-05
Hepatitis C	KEGG	2.44E-08	1.25E-05
Cytosolic DNA-sensing pathway	KEGG	9.71E-08	4.63E-05
TRAF3-dependent IRF activation pathway	REACTOME	2.17E-07	9.71E-05
Innate Immune System	REACTOME	3.08E-07	1.30E-04
NOD-like receptor signaling pathway	KEGG	3.55E-07	1.41E-04
Influenza A	KEGG	4.23E-07	1.59E-04
Interferon Signaling	REACTOME	1.96E-06	6.68E-04
Signal regulatory protein (SIRP) family interactions	REACTOME	4.17E-06	1.35E-03
Factors involved in megakaryocyte development and platelet production	REACTOME	1.16E-05	3.33E-03
Adaptive Immune System	REACTOME	1.25E-05	3.45E-03
Activation of IRF3/IRF7 mediated by TBK1/IKK epsilon	REACTOME	1.72E-05	4.40E-03
Ovarian tumor domain proteases	REACTOME	1.87E-05	4.60E-03
Signaling by Interleukins	REACTOME	2.05E-05	4.88E-03
Osteoclast differentiation	KEGG	2.18E-05	5.03E-03
NF-kappa B signaling pathway	KEGG	2.27E-05	5.08E-03
Natural killer cell mediated cytotoxicity	KEGG	2.57E-05	5.57E-03
Toll Like Receptor 3 (TLR3) Cascade	REACTOME	2.98E-05	5.91E-03
MyD88-independent TLR3/TLR4 cascade	REACTOME	2.98E-05	5.91E-03
TRIF-mediated TLR3/TLR4 signaling	REACTOME	2.98E-05	5.91E-03
Intestinal immune network for IgA production	KEGG	7.49E-05	1.28E-02
Activated TLR4 signalling	REACTOME	7.78E-05	1.29E-02
Epstein-Barr virus infection	KEGG	8.80E-05	1.43E-02
Toll Like Receptor 4 (TLR4) Cascade	REACTOME	1.38E-04	2.10E-02
Hemostasis	REACTOME	1.87E-04	2.57E-02
Ras signaling pathway	KEGG	2.05E-04	2.75E-02
Tuberculosis	KEGG	2.08E-04	2.75E-02
Signaling by the B Cell Receptor (BCR)	REACTOME	2.74E-04	3.56E-02

Negative regulators of RIG-I/MDA5 signaling	REACTOME	2.93E-04	3.74E-02
Primary immunodeficiency	KEGG	3.26E-04	3.89E-02
T cell receptor signaling pathway	KEGG	3.26E-04	3.89E-02
Downstream signaling events of B Cell Receptor (BCR)	REACTOME	4.26E-04	4.99E-02
B cell receptor signaling pathway	KEGG	4.37E-04	4.99E-02

The statistics are provided by ToppGene (https://toppgene.cchmc.org/). P-value is two-sided. FDR is the P-value after adjusting multiple testing.

Supplementary Table 4 Groups of putative SLE genes based on heterogeneity in effect-size estimates

Category	#Loci	#Genes	Genes identified at the disease loci
Ancestry-shared loci with CQ-test *P* > 0.05 (**Category 1**)	79	120	SDF4,B3GALT6,FCGR2A,LOC101928673,KIAA0040,RABGAP1L,PTPRC,IKBKE,MAPKAPK2,LYST,INPP5B,MTF1,IL12RB2,RERE,WDFY4,ARID5B,ZNF365,TREH,ETS1,PDHX,IRF7,UNC119B,COQ5,MLEC,CABP1,SLC15A4,TM9SF2,SETDB2,ARL11,TRAF3,RASGRP1,SCAMP2,ULK3,PPCDC,TBC1D2B,HMG20A,CLEC16A,CCL22,CCL17,PRSS54,CSNK2A2,ZFP90,IRF8,GRB2,MIF4GD,GGA3,SLC25A19,CD226,KIAA1683,LRRC25,ANKRD27,CD37,FLT3LG,IRF3,RRAS,FCGRT,ALDH16A1,SIGLEC6,PPP6R1,TMEM86B,ARHGAP15,IFIH1,GCA,NAB1,TMEM194B,MFSD6,COQ10B,CTLA4,YPEL5,LINC01936,TET3,SIRPB2,SIRPG,SIRPB1,SIRPD,CD40,NCOA5,UBE2L3,CD80,ARHGAP31,POGLUT1,IL12A-AS1,ACTRT3,LPP,AC098973.1 ,PXK,BANK1,CLNK,TNIP2,RNF4,GAK,IDUA,TERT,TCF7,UBE2B,CDKN2AIPNL,TNIP1,MIR3142HG,IL7R,CAPSL,RP11-79C6.3,SPEF2,PRDM1,UHRF1BP1,BACH2,ZPBP,STAG3L4,NCF1,BLK,LINC00208,C8orf12,RP11-89M16.1,MFHAS1,ERI1,NR4A3,IFNA21,IFNA7,IFNB1,IFNA2,IFNA5
Putative ancestry-heterogenous loci with CQ-test *P* < 0.05 and FDR adjusted CQ-test *P* > 0.05 (**Category 2**)	20	22	CD58,AL390066.1,TNFSF4,RGS1,IL10,DDX6,SIPA1,RELA,MAP3K11,PCNXL3,GPR19,RAD51B,STAT4,IKZF2,RASGRP3,SPRED2,HIC2,PDGFB,TNFAIP3,IRF5,CASC11 ,NCOA2
Ancestry-heterogenous loci with adjusted CQ-test *P* < 0.05 (**Category 3**)	9	9	PRKCB,TYK2,PLD4,TNFRSF13B,IKZF1,HIP1,DSE,ACAP1,PRRX1
Disease loci with risk allele monomorphic in one of the two ancestries (**Category 4**)	7	12	PTPN22,BCL2L15,NCF2,SH2B3,TRAFD1,PTPN11,TNFSF13B,IKZF3,GSDMB,ORMDL3,ATG16L2, IGHG1

Supplementary Table 5 SLE-associated loci with heterogenity between East Asian and European populations

SNP	Chr	Pos	Risk allele	GENE	Function	East Asian population				European population				Cochran's Q-test
						Risk allele freq	OR	SE	P	Risk allele freq	OR	SE	P	
rs12132445	1	170811799	A	PRRX1-MROH9	intergenic	0.616	**1.17**	0.03	6.75E-08	0.458	**1.00**	0.03	9.67E-01	1.05E-04
rs9387400	6	116694120	C	DSE	intronic	0.052	**1.35**	0.05	3.14E-08	0.627	**1.06**	0.03	2.96E-02	1.17E-04
rs4917014	7	50305863	T	IKZF1	intergenic	0.709	**1.33**	0.03	5.18E-29	0.679	**1.16**	0.03	1.34E-06	4.02E-04
rs11773745	7	75171438	A	HIP1	intronic	0.676	**1.24**	0.03	2.52E-11	0.266	**1.00**	0.03	8.55E-01	3.77E-06
rs2841280	14	105393556	C	PLD4	missense	0.575	**1.21**	0.03	6.12E-11	0.468	**1.05**	0.03	7.10E-02	5.15E-04
rs4592664	16	23890735	T	PRKCB	intronic	0.766	**1.17**	0.03	2.37E-06	0.804	**0.96**	0.03	1.90E-01	2.23E-05
rs9899849	17	7234983	A	NEURL4-ACAP1	intergenic	0.293	**0.97**	0.03	3.32E-01	0.225	**1.21**	0.03	1.10E-08	1.72E-06
rs34562254	17	16842991	A	TNFRSF13B	missense	0.455	**1.18**	0.03	2.88E-08	*0.107*	**1.01**	0.04	7.57E-01	3.58E-04
rs2304256	19	10475652	C	TYK2	missense	0.427	**0.99**	0.02	7.58E-01	0.713	**1.23**	0.03	8.12E-11	2.25E-07

Risk allele frequency in East Asians is estimated by using the 3,324 HK controls, and the risk allele frequency in Europeans is estimated by using the 5,379 controls in the EUR GWAS 2 cohort.

Supplementary Table 6 Twenty-seven immune-unrelated phenotypes studied in European populations used to compare with East Asian SLE association signals in colocalization analyses

Index	Traits	Consortium
1	Coronary Artery Disease (CAD)	CARDIoGRAM
2	Forearm bone mineral density (FABMD)	GEFOS
3	Femoral neck bone mineral density (FNBMD)	GEFOS
4	Lumbar spine bone mineral density (LSBMD)	GEFOS
5	Extreme BMI	GIANT
6	Extreme Height	GIANT
7	Extreme waist hip ratio (WHR)	GIANT
8	HDL	GLGC
9	LDL	GLGC
10	Total Cholesterol (TC)	GLGC
11	Triglycerides (TG)	GLGC
12	Fasting Glucose (FG)	MAGIC
13	Acetoacetate	MAGNETIC
14	Acetate	MAGNETIC
15	Alanine	MAGNETIC
16	Albumin	MAGNETIC
17	Anorexia	PGC
18	Autism Spectrum Disorder (ASD)	PGC
19	Bipolar Disorder (BIP)	PGC
20	Major depressive disorder (MDD)	PGC
21	Schizophrenia	PGC
22	Educational attainment	SSGAC
23	Neuroticism	SSGAC
24	Insomnia	SSGAC
25	Smoking/cigarettes per day	TAG
26	Aging	Aging
27	Childhood obesity	EGG
