# Supplementary material for: Identification of 38 novel loci for systemic lupus erythematosus and genetic heterogeneity between ancestral groups
Source: Nat Commun. 2021 Feb 3;12:772. doi: 10.1038/s41467-021-21049-y (PMC7858632; doi:10.1038/s41467-021-21049-y)
Supplement: Supplementary file 2 — Reporting Summary [file 41467_2021_21049_MOESM2_ESM.pdf]

nature research

Corresponding author(s): Dr. Yang Wanling
Last updated by author(s): Dec 10, 2020

Reporting Summary

Nature Research wishes to improve the reproducibility of the work that we publish. This form provides structure for consistency and transparency in reporting. For further information on Nature Research policies, see our Editorial Policies and the Editorial Policy Checklist.

Statistics

For all statistical analyses, confirm that the following items are present in the figure legend, table legend, main text, or Methods section.

n/a	Confirmed	
☐	☒	The exact sample size (*n*) for each experimental group/condition, given as a discrete number and unit of measurement
☐	☒	A statement on whether measurements were taken from distinct samples or whether the same sample was measured repeatedly
☐	☒	The statistical test(s) used AND whether they are one- or two-sided<br>*Only common tests should be described solely by name; describe more complex techniques in the Methods section.*
☐	☒	A description of all covariates tested
☐	☒	A description of any assumptions or corrections, such as tests of normality and adjustment for multiple comparisons
☐	☒	A full description of the statistical parameters including central tendency (e.g. means) or other basic estimates (e.g. regression coefficient) AND variation (e.g. standard deviation) or associated estimates of uncertainty (e.g. confidence intervals)
☐	☒	For null hypothesis testing, the test statistic (e.g. *F*, *t*, *r*) with confidence intervals, effect sizes, degrees of freedom and *P* value noted<br>*Give P values as exact values whenever suitable.*
☒	☐	For Bayesian analysis, information on the choice of priors and Markov chain Monte Carlo settings
☒	☐	For hierarchical and complex designs, identification of the appropriate level for tests and full reporting of outcomes
☐	☒	Estimates of effect sizes (e.g. Cohen's *d*, Pearson's *r*), indicating how they were calculated

Our web collection on statistics for biologists contains articles on many of the points above.

Software and code

Policy information about availability of computer code

Data collection	Illumina GenomeStudio 2.0 was used to perform genotyping for individuals and transformed the results into PLINK format.
Data analysis	PLINK (v1.90) was used to perfom quality control procedures and PCA analysis. Genotype Harmonizer (version 1.4.20) was used to align the strands of variants with the reference of 1,000 Genomes Project. SHAPEIT (v2.r837) was used for pre-phasing , IMPUTE2 (v2.3.2) was used for the genotype imputation and SNPTEST (v2.5.4) was used for association study. Popcorn (version 0.9.6) was perfomed for transethnic genetic-correlation estimates. LDSC regression (v1.0.0) was used for functional enrichment analysis. DEPICT (v1.1 beta) was used for prioritizing disease genes and ToppGene (2019 version) for gene set enrichment analysis. PAINTOR (version 3.0) was used for fine-mapping analysis and R package Coloc (v3.1) was used to evaluate the probability of associaition cocloclation. R package Rehh (v2.0.4) was used to estimate Integrated Haplotype Score across different ancestries. Lassosum (version 0.4.4) and LDpred (version 1.0.6) were used for polygenic risk score calculation. The area under the ROC curve (AUC) and the optimal cutoff were calculated using the R package pROC (v1.13.0). RELI (Jan 30, 2019) was used to identify enriched transcription binding sites among the disease-associated loci. VarExplained (Mar 2011) was used to calculate the variance in liability explained by the associated variants.

For manuscripts utilizing custom algorithms or software that are central to the research but not yet described in published literature, software must be made available to editors and reviewers. We strongly encourage code deposition in a community repository (e.g. GitHub). See the Nature Research guidelines for submitting code & software for further information.

Data

Policy information about availability of data

All manuscripts must include a data availability statement. This statement should provide the following information, where applicable:

- Accession codes, unique identifiers, or web links for publicly available datasets
- A list of figures that have associated raw data
- A description of any restrictions on data availability

Genome-wide association summary statistics for the East Asian populations can be accessed through the GWAS Catalog (GCST90011866). The data for the European populations are available at http://insidegen.com/ and http://urr.cat/data/GWAS_SLE_summaryStats.zip. The ImmunoChip data are publicly available for download at https://www.ncbi.nlm.nih.gov/pmc/articles/PMC4767573/bin/NIHMS747721-supplement-3.xlsx. Summary association statistics for other phenotypes are downloaded from LD hub (http://ldsc.broadinstitute.org/). Summary statistics for eQTL results are retrieved from Blood eQTL browser (https://genenetwork.nl/bloodeqtlbrowser/). Protein-protein interaction information is downloaded from STRING database (https://string-db.org/). Histone modifications across cell types are downloaded from the Roadmap Epigenomics Project (http://www.roadmapepigenomics.org/).

Field-specific reporting

Please select the one below that is the best fit for your research. If you are not sure, read the appropriate sections before making your selection.

☒ Life sciences ☐ Behavioural & social sciences ☐ Ecological, evolutionary & environmental sciences

For a reference copy of the document with all sections, see nature.com/documents/nr-reporting-summary-flat.pdf

Life sciences study design

All studies must disclose on these points even when the disclosure is negative.

Sample size	In total, ten SLE genetic cohorts consisting of 11,283 cases and 24,086 controls were involved in this study. This is one of the largest association studies on SLE. The much-increased sample size provides adequate power to detect novel disease susceptibility loci and to compare differences between East Asians and European populations.
Data exclusions	We have removed individuals with poor genotyping, DNA contamination, potential relationships and unmatched ancestral background. Individuals would be removed based on the following pre-established criteria: i) missing genotypes (>5%), ii) hidden relatedness (identity-by-descent > 12.5%), iii) inbreeding coefficients (>0.05 or < -0.05) and iv) having PC outliers (population stratification). These criteria are all well-established and widely used in GWAS. The rationale is to try to eliminate false positive associations that can be caused by genotyping errors (poor genotyping quality, sample contamination) and by mismatch between cases and controls (PC outliers) and to ensure they are comparisons between affected and unaffected unrelated individuals from the same population (relationship check, inbreeding coefficient).
Replication	Of the ten SLE genetic cohort, cohort from GZ is newly generated and more samples were included in the HK and CC GWAS cohorts. The four SLE GWAS cohorts from European populations and summary statistics from Immunochip studies of East Asians were published before, and we included these data as replication for detecting new disease-assocaited loci.
Randomization	Randomization refers to randomly assigning participants to treatment or placebo groups. This is not applicable to genetic association studies, which are comparisons of allele frequencies of genetic variants between affected and unaffected individuals collected in a cohort. There is not a selection and assigning process involved in association studies.
Blinding	For the same reason to the question of ‘randomization’, blinding is not applicable to genetic association studies.

Reporting for specific materials, systems and methods

We require information from authors about some types of materials, experimental systems and methods used in many studies. Here, indicate whether each material, system or method listed is relevant to your study. If you are not sure if a list item applies to your research, read the appropriate section before selecting a response.

Materials & experimental systems

n/a	Involved in the study
☒	☐ Antibodies
☒	☐ Eukaryotic cell lines
☒	☐ Palaeontology and archaeology
☒	☐ Animals and other organisms
☐	☒ Human research participants
☒	☐ Clinical data
☒	☐ Dual use research of concern

Methods

n/a	Involved in the study
☒	☐ ChIP-seq
☒	☐ Flow cytometry
☒	☐ MRI-based neuroimaging

April 2020

Human research participants

Policy information about studies involving human research participants

Population characteristics	The subjects involved in this study include Hong Kong Han Chinese, Han Chinese population living in Guangzhou, and Han Chinese population living in central China. As germline variants at the autosomes are unlikely to be affected by sex, age and other epidemiological factors, we didn't include epidemiological factors as co-variates in the genome-wide association analysis. This practice is widely accepted in genome-wide association studies.
Recruitment	All SLE cases involved in this study have medical records documenting fulfillment of the revised criteria of the American College of Rheumatology for diagnosis of SLE. Controls for the HK cohort are healthy blood donors from the Hong Kong Red Cross and HK residents who participated in other immune-unrelated GWAS. Controls for the GZ cohort are healthy blood donors and individuals lived in GZ who participated in other immune-unrelated GWAS. Controls for the CC cohort are pool of healthy blood donors lived in Hefei and Shanghai.<br>In our study, more than 98% of the individuals who were invited to join this study chose to participate in our study. Thus, it is unlikely that self-selection bias would be an issue in our study. Replication of results from multiple independent cohorts also ensures the validity of the results.
Ethics oversight	The University of Hong Kong, Hospital Authority Hong Kong West Cluster and Guangzhou Women and Children's Medical Center approved the study.

Note that full information on the approval of the study protocol must also be provided in the manuscript.
