# Supplementary material for: Identification of 38 novel loci for systemic lupus erythematosus and genetic heterogeneity between ancestral groups
Source: Nat Commun. 2021 Feb 3;12:772. doi: 10.1038/s41467-021-21049-y (PMC7858632; doi:10.1038/s41467-021-21049-y)
Supplement: Supplementary file 3 — Description of Additional Supplementary Files [file 41467_2021_21049_MOESM3_ESM.pdf]

Description of Additional Supplementary Files

Supplementary Data 1 Summary association statistics of reported SLE-associated variants

Supplementary Data 2 Summary association statistics of 38 novel SLE susceptibility loci

Supplementary Data 3 Transcription factors whose binding sites are enriched in the SLE-associated loci

Supplementary Data 4 Putative disease genes identified at each associated locus

Supplementary Data 5 Summary of fine-mapping results based on ancestry-dependent and trans-ancestral analyses
